# Lipid storage myopathy associated with sertraline treatment is an acquired mitochondrial disorder with respiratory chain deficiency

**DOI:** 10.1007/s00401-024-02830-x

**Published:** 2024-11-26

**Authors:** Carola Hedberg-Oldfors, Ulrika Lindgren, Kittichate Visuttijai, Yan Shen, Andreea Ilinca, Sara Nordström, Christopher Lindberg, Anders Oldfors

**Affiliations:** 1 Department of Laboratory Medicine, University of Gothenburg, Gothenburg, Sweden; 2 Neuromuscular Centre, Department of Neurology, Sahlgrenska University Hospital, Gothenburg, Sweden; 3 Department of Neurology, Division of Neurology, Skåne University Hospital, Lund, Sweden; 4 Department for Clinical Sciences, Lund University, Lund, Sweden

**Keywords:** Lipid storage myopathy, Muscle weakness, SSRI, Sertraline, Metabolic crisis, Multiple acyl-CoA dehydrogenase deficiency

## Abstract

Lipid storage myopathies are considered inborn errors of metabolism affecting the fatty acid metabolism and leading to accumulation of lipid droplets in the cytoplasm of muscle fibers. Specific diagnosis is based on investigation of organic aids in urine, acylcarnitines in blood and genetic testing. An acquired lipid storage myopathy in patients treated with the antidepressant drug sertraline, a serotonin reuptake inhibitor, has recently emerged as a new tentative differential diagnosis. We analyzed the muscle biopsy tissue in a group of 11 adult patients with muscle weakness and lipid storage myopathy which developed at a time when they were on sertraline treatment. This group comprise most patients with lipid storage myopathies in western Sweden during the recent nine-year period. By enzyme histochemistry, electron microscopy, quantitative proteomics, immunofluorescence of the respiratory chain subunits, western blot and genetic analyses we demonstrate that muscle tissue in this group of patients exhibit a characteristic morphological and proteomic profile. The patients also showed an acylcarnitine profile in blood suggestive of multiple acyl-coenzyme A dehydrogenase deficiency, but no genetic explanation was found by whole genome or exome sequencing. By proteomic analysis the muscle tissue revealed a profound loss of Complex I subunits from the respiratory chain and to some extent also deficiency of Complex II and IV. Most other components of the respiratory chain as well as the fatty acid oxidation and citric acid cycle were upregulated in accordance with the massive mitochondrial proliferation. The respiratory chain deficiency was verified by immunofluorescence analysis, western blot analysis and enzyme histochemistry. The typical ultrastructural changes of the mitochondria included pleomorphism, dark matrix and frequent round osmiophilic inclusions. Our results show that lipid storage myopathy associated with sertraline treatment is a mitochondrial disorder with respiratory chain deficiency and is an important differential diagnosis with characteristic features.

**Supplementary Information:**

The online version contains supplementary material available at 10.1007/s00401-024-02830-x.

## Introduction

**Fig. 1:**
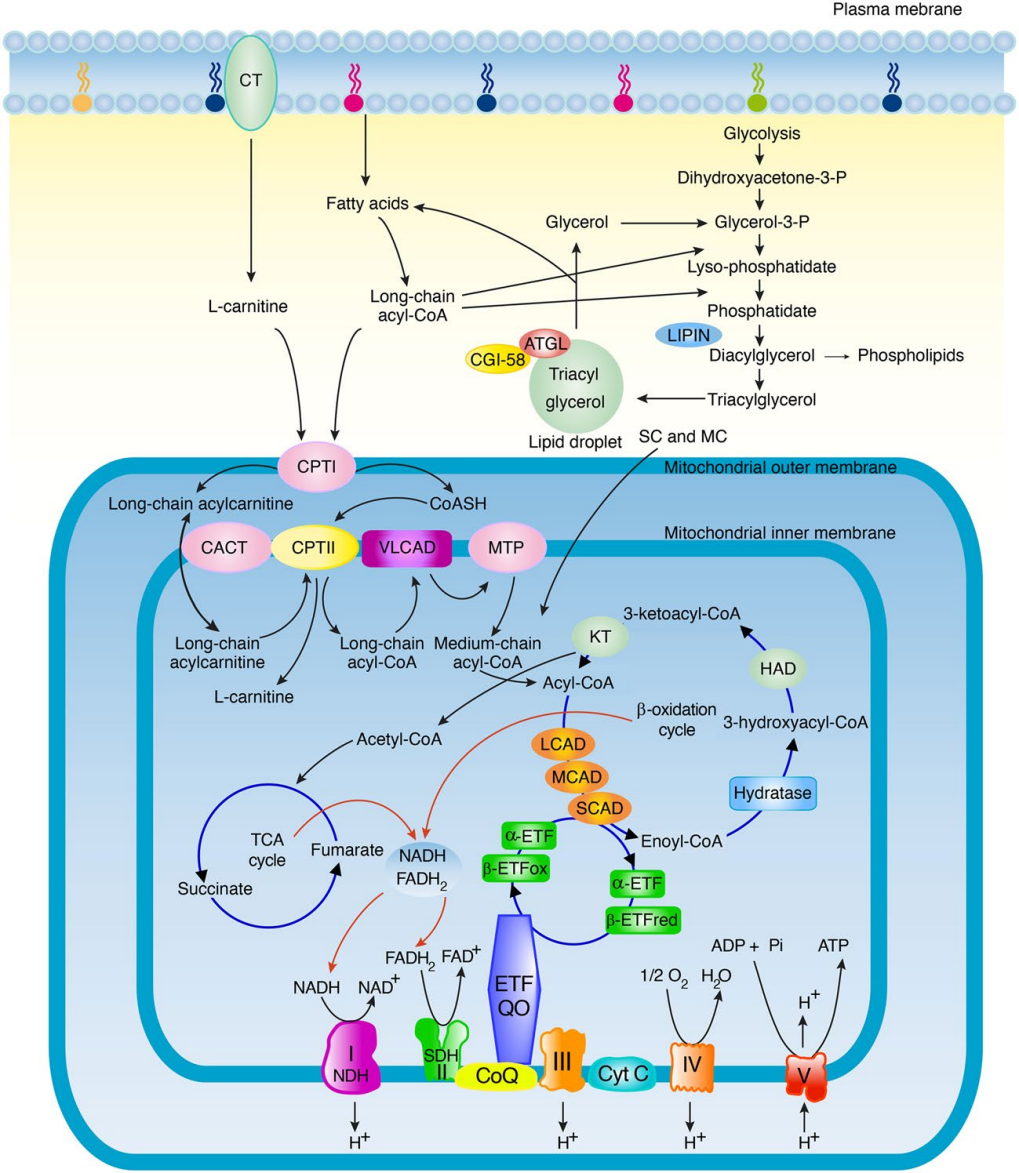
Schematic illustration of the fatty acid metabolism. Lipid storage is located in cytoplasmic lipid droplets. Carnitine and carnitine palmitoyl transferase (CPT) I and II are involved in transport of long chain fatty acyl-CoA into the mitochondria. Short-chain acyl-CoA (SC) and medium-chain acyl-CoA (MC) directly diffuse into mitochondria. In the β-oxidation cycle acyl-CoA is metabolized by different acyl-CoA dehydrogenases to create acetyl-CoA entering the citric acid cycle (TCA). NADH and FADH_2_ are substrates entering the respiratory chain composed of five Complexes (I–V). Complex II is equivalent to succinate dehydrogenase (SDH) and Complex IV is equivalent to cytochrome c oxidase (COX). In the final step ATP is formed by phosphorylation of ADP by Complex V (ATP synthase) utilizing the energy from the proton gradient that has been built up across the inner mitochondrial membrane by Complex I, II and IV. The ETF-coenzyme Q oxidoreductase (ETF:CQ) catalyzes the transfer of electrons from electron transferring flavoprotein (ETF) to ubiquinone, reducing it to ubiquinol. CT; carnitine transporter, ATGL, adipose triglyceride lipase; CGI-58, activator of ATGL; CACT; carnitine/acylcarnitine translocase, VLCAD, LCAD, MCAD, SCAD, very long-, long-, medium- and short chain acyl-CoA dehydrogenase, respectively; MTP, mitochondrial trifunctional protein; Hydratase, 2-enoyl-CoA hydratase; HAD, L-3-hydroxyacyl-CoA dehydrogenase; KT, 3-ketoacyl-CoA thiolase; respiratory chain Complex I (NDH, NADH: coenzyme Q reductase); CoQ, coenzyme Q; Cyt C, cytochrome c

Lipid storage myopathies is a group of rare metabolic disorders characterized by an abnormal accumulation of lipids within muscle fibers. They are associated with various signs and symptoms such as muscle weakness, exercise intolerance, myalgia, rhabdomyolysis, and metabolic crisis. Lipid storage myopathies can be caused by genetic variants in genes that encode enzymes involved in muscle fatty acid metabolism (Fig. 1) and include among others neutral lipid storage disease, lipin-1 deficiency, carnitine deficiency, and multiple acyl-CoA dehydrogenase deficiency (MADD) [2]. Abnormal lipid storage in muscle fibers may also occur in primary disorders of the mitochondrial respiratory chain [10]. Depending on the affected enzyme, acylcarnitines of various lengths are frequently elevated in blood. The acylcarnitine profile is an important diagnostic tool that may direct the search for variants in specific genes involved in the β-oxidation of fatty acids [8, 24]. MADD is frequently responsive to riboflavin treatment and is often diagnosed based on the acylcarnitine profile in blood [2, 8].

The diagnostic work-up of lipid storage myopathy in adults frequently results in absence of a clear genetic diagnosis. Recently it was demonstrated that treatment with sertraline, an antidepressant drug, may be involved in the pathogenesis of lipid storage myopathy [26]. Most of the patients also showed a MADD-like blood acylcarnitine profile and responded to riboflavine treatment.

We retrospectively analyzed the patients with lipid storage myopathy at the neuropathology laboratory in Gothenburg from 2015 to 2023 and found that only a few had a genetic diagnosis in spite of a MADD-like blood acylcarnitine profile. Nearly all the patients without a genetic diagnosis had been on sertraline treatment at the time of onset of muscle weakness. In this study, we have investigated the muscle-biopsy specimens from these patients by applying morphological, proteomic and genetic analyses. We demonstrate that lipid storage myopathy associated with sertraline treatment shows a characteristic mitochondrial pathology with profound respiratory chain deficiency mainly affecting Complex I, II, and IV.

## Materials and methods

### Patients and medical records

Seventeen adult patients diagnosed with lipid storage myopathy based on muscle biopsy at the neuromuscular center in Gothenburg during a 9-year period from 2015–2023 were identified. Two of them had a clear genetic diagnosis whereas in 15 no genetic diagnosis had been identified by whole genome (WGS) or whole exome sequencing (WES). After approval by the Swedish Ethical Review Authority, 12 of the patients without genetic diagnosis gave their written informed consent to participate in the study. Eleven patients had been on sertraline treatment at the time of investigation for their muscle weakness, and were further studied. Clinical and laboratory data are summarized in Table 1. All 11 patients had been investigated for acylcarnitines in blood by mass spectrometry analysis (Supplementary Table 1).

**Table 1:** Summary of clinical and laboratory data

Patient	11
Gender (female/male)	7/4
Age at symptoms onset (y)	Median 48, range 22–62
Sertraline treatment before symptoms onset (y)	Median 3, range 0,5- > 20
Age at biopsy (y)	Median 50, range 23–64
Sertraline treatment at biopsy#	11^#^/11
Dose sertraline at biopsy (mg, 1 × 1)	Median 150, range 50–300
CK elevated	4/11
EMG, Myopathic	7/11
Proximal muscle weakness	11/11
Distal muscle weakness	8/11
Neck muscle weakness	6/11
Dysphagia	3/11
Myalgia	9/11
Sensory disturbance	7/11
Elevated acylcarnitines in blood	9/11
Negative genetic screening	11/11

^#^ In 1 patient sertraline was discontinued 3 weeks before the diagnostic biopsy

y = years

*CK* creatine kinase. First available value after symptom onset

*EMG* electromyography

### Muscle biopsy and histochemistry

Open skeletal muscle biopsy was performed in all patients. Specimens were mounted on cork plates, snap-frozen in isopentane cooled by liquid nitrogen and stored at −80°C. Control skeletal muscle tissue included anonymized muscle-biopsy specimens from age and sex-matched individuals who had been investigated for a possible muscle disorder, but in whom the clinical and histopathological investigations excluded muscle disease (Supplementary Table 2).

Standard techniques were applied for histochemical analyses of the muscle-biopsy specimens [10]. For muscle fiber typing, a quadruple immunofluorescence method was applied as previously described [13]. This method is based on myosin heavy-chain expression and allows for muscle fiber typing (type 1, type 2A, type 2B and hybrid muscle fibers) in one single section. Antibodies used in this assay are listed in Supplementary Table 3. For analysis of the mitochondrial respiratory chain complexes I, II and IV, a quadruple immunofluorescence method (MITIF) was applied [1]. The method allows for simultaneous investigation of two of the complexes and mitochondrial membranes in a single section. For detailed methods of this assay, as well as illustrations of defined disease control specimens see Supplementary material.

### Genetic analysis

Total genomic DNA was isolated from muscle-biopsy specimens using standard protocols. The DNA was subjected to WES or WGS according to manufacturers’ protocols (Illumina, San Diego, CA, USA). The paired-end reads were aligned to the reference genome (hg19) and the mitochondrial DNA (mtDNA) (NC_012920.1). Variants were called and filtered for identification of potentially pathogenic variants in candidate genes associated with myopathy and or metabolic disorder based on The Gene Table of Neuromuscular Disorders 2024 [4] (www.musclegenetable.fr/), MitoCarta 3.0 [21] and mtDNA (https://www.mitomap.org/MITOMAP). Some genes associated with lipid storage myopathy and MADD-like acylcarnitine profile were analyzed in more detail (*PNPLA2*, *HADHA*, *HADHB*, *ETFA*, *ETFB*, *ETFDH*, *COASY*, *FLAD1*, *ACADS*).

Large mtDNA deletions and duplications were analyzed using the WGS data by an in-house bioinformatic tool, MitoSAlt, as previously described [3], except that mean coverage depth was based on the entire mtDNA.

Copy number of mtDNA in relation to nuclear DNA was estimated as previously described: mtDNA copy number = mitochondrial genome coverage × 2/nuclear genome coverage [3, 14].

### Quantitative mass spectrometry

Skeletal muscle protein extracts from eight patients and eight normal controls were prepared from fresh frozen muscle biopsies. For quantitative analysis the proteins were labelled using TMTpro 18-plex isobaric mass tagging reagents (Thermo Fischer Scientific) and analyzed by nanoscale liquid chromatography-tandem mass spectrometry (LC-MS^3^) according to details described in Supplementary material.

### Statistical analyses

The data were log2-transformed, and then, for each protein, log2 fold change (log2FC) and *p* values were computed using Welch’s *t* test for patients versus controls. To control for multiple comparisons, the Benjamini–Hochberg method was used to adjust the *p* values, and proteins with a false discovery rate (FDR) less than 0.05 were considered significant for further analysis. Zero abundance values were replaced with half the detection limit (LOD/2).

### Western blot analysis

Western blot analyses were performed on proteins extracted from skeletal muscle tissue and performed as previously described [23] (see Supplementary Table 3 for antibodies used and more details). Coomassie staining (SimplyBlue SafeStain, LC6060, Invitrogen) of myosin heavy chain (MHC) served as loading control.

## Results

### Muscle histochemistry

**Fig. 2:**
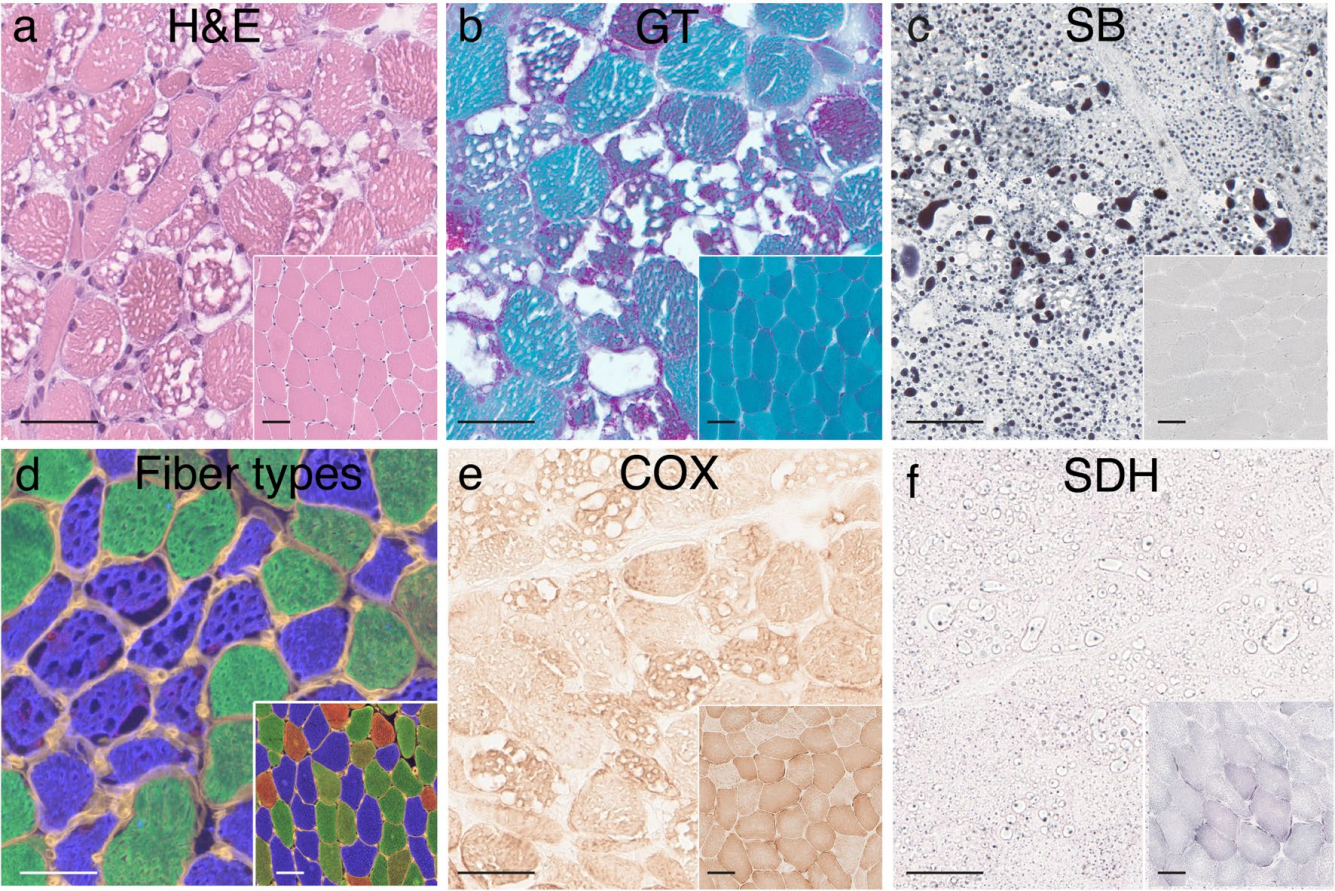
Muscle pathology in patient P10. Insets are normal controls. **a** Multiple large and small vacuoles are present in the muscle fibers (hematoxylin and eosin, H&E) **b** In Gomori trichrome (GT) staining, mitochondrial proliferation is seen as red granular material, mainly in fibers with vacuoles. **c** Lipid staining (Sudan black, SB) showing the storage material in the vacuolated fibers to be composed of lipids. **d** Muscle fiber typing by immunofluorescence analysis of myosin heavy-chain isoforms showing that type 1 (blue) muscle fibers are more vacuolated that type 2A (green) muscle fibers. Type 2B (red) muscle fibers are only present in the control (inset). **e** Enzyme histochemical staining of cytochrome c oxidase (COX) showing variable and in general pale staining of muscle fibers indicating a partial COX deficiency. **f** Enzyme histochemical staining of succinate dehydrogenase (SDH) showing pale (and partially artefactual staining due to lipid accumulation) indicating SDH deficiency. Bars = 50 µm

All patients had a vacuolar myopathy due to lipid storage (Fig. 2a–b). The amount of lipid varied from moderately increased to massive accumulation as seen by lipid staining with Sudan black (Fig. 2c, Table 2). In general, the type 1 muscle fibers were more affected with larger vacuoles (Fig. 2d). Enzyme histochemical staining for oxidative enzymes (Complex II; succinate dehydrogenase, SDH, and Complex IV; cytochrome c oxidase, COX) showed a reduced staining intensity in most patients compared to controls (Fig. 2e–f, Table 2). Gomori trichrome (GT) staining (Fig. 2b) and electron microscopy (see below) showed a marked increase in the number of mitochondria. Typical ragged red muscle fibers were infrequent. In some of the cases occasional scattered necrotic fibers were observed (Table 2). There was no apparent increase in interstitial connective tissue.

**Table 2:** Detailed clinical and laboratory findings

Patient	1	2	3	4	5	6	7	8	9	10	11
Gender (F; female/M, male)	M	F	M	M	F	F	F	M	F	F	F
Age at symptoms onset (years)	44	54	48	54	59	45	23	61	23	29	62
Sertraline treatment before symptom onset (years)	10	1–2	3	6	0–1	9	1,5	0,5	7	>1,5	>20
Age at biopsy (years)	46	56	50	55	59	45	23	61	26	34	64
Dose sertraline at biopsy (mg, 1 × 1)	100	100	150^#^	300	50	100	200	200	150	200	150
**Muscle biopsy investigations**											
*Histochemistry*											
Necrotic fibers			+	+		+	+			+	
Mitochondrial proliferation	+	++	+++	+++	+	+++	+++	+	+	+++	+++
Lipid storage	++	++	+++	+++	+	++	+++	++	+	+++	+++
*Electron microscopy*											
Clusters of lipid droplets	++	+++	+++	+++	nd	nd	nd	nd	+	+++	+++
Mitochondrial proliferation	+	+++	+++	+++	nd	nd	nd	nd	++	+++	+++
Predominantly small dark mitochondria	yes	yes	yes	no	nd	nd	nd	nd	yes	yes	no
Mitochondrial inclusions	yes	yes	yes	yes	nd	nd	nd	nd	no	yes	yes
*MITIF*											
Complex I deficiency	+++	+++	+++	+++	+	+++	+++	+++	++	+++	++
Complex II deficiency		++	+	++		++	++	++	+	++	+
Complex IV deficiency	++	++	++	++		+	+	+	+	++	++
*Enzyme histochemistry*											
SDH deficiency (weak staining)				+		+	+	+	+	+	+
COX deficiency (weak staining)				+				+			
*Western blot (amount of protein)*											
Complex I (NDUFB8)	↓	↓	(↓)	↓		↓	↓	↓		↓	↓
Complex II (SDHB)		(↓)	(↓)	(↓)		(↓)	(↓)	(↓)		↓	
Complex III (UQCRC2)											
Complex IV (MTCO1)	↓		(↓)	(↓)		(↓)	(↓)	↓		(↓)	(↓)
Complex V (ATPB)											
VDAC1	↑	↑	↑	↑	↑		↑		↑	↑	↑
mtDNA copy number*	8291	12,178	nd	8297	7293	6235	4911	3314	6368	8748	11,376
*Proteomic analysis by LC-MS*^*3*^	+	+	+	+	nd	+	+	nd	nd	+	+

^#^, discontinued 3 weeks before the diagnostic biopsy

Muscle-biopsy investigations: +, ++, +++ = level of alterations from occasional/slight (+) to extensive (+++)

MITIF; Mitochondrial immunofluorescence, +, ++, +++ = different levels of deficiency from slight to severe

Enzyme histochemistry, + = weak staining is present

Western blot: ↓ down-regulated, ↑ up-regulated, (↓) slightly down-regulated

*nd* not done

^*^, calculated from WGS data using MitoSAlt

### Electron microscopy

**Fig. 3:**
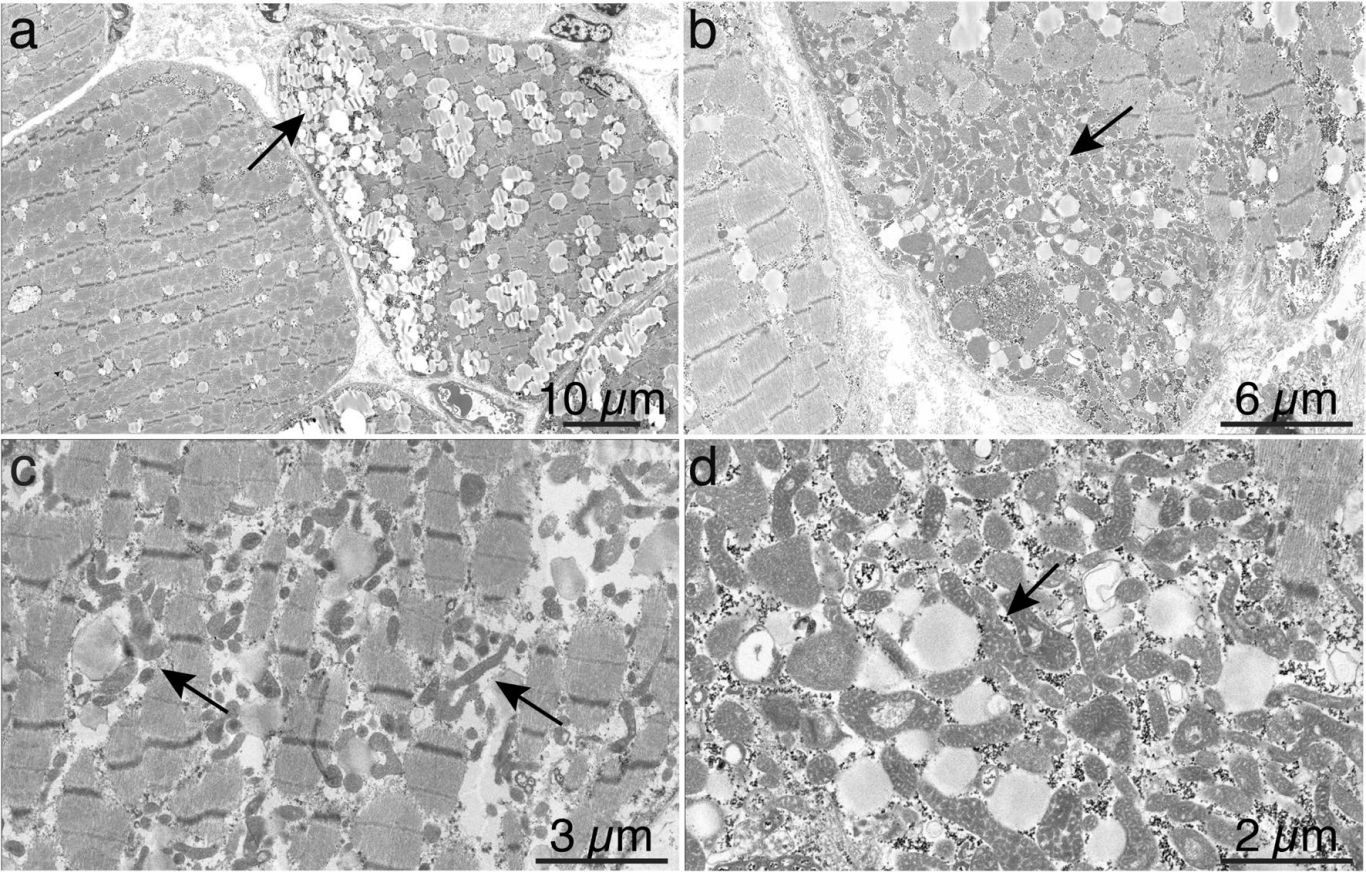
Electron micrograph of muscle in patient P10. **a** Abnormal lipid storage in the muscle fibers is seen as increased size of lipid droplets, which in some fibers show clustering (arrow). **b** There is an abnormally large number of pleomorphic mitochondria, which in this image cluster in a subsarcolemmal region. **c** In this fiber the pleomorphic, dark mitochondria are accumulated in the intermyofibrillar compartment (arrows). **d** At high magnification the dense matrix of the mitochondria is seen, as well as their close connection to lipid droplets (arrow)

Electron microscopy was performed in seven of the 11 patients. All patients showed mitochondrial proliferation, which was extensive in some patients (Fig. 3, Table 2). Most of the patients displayed numerous small mitochondria with dark matrix resulting in an overall dark appearance of the mitochondria. Although many of the mitochondrial profiles appeared unusually small, many were elongated (Fig. 3 and Supplementary Fig. 2 and 3). In several patients, some enlarged mitochondria measuring more than 1 µm in diameter were observed (Fig. 3b and Supplementary Fig. 2 and 3). The arrangements of mitochondrial cristae were in general not deranged, but in most cases small electron-dense inclusions, frequently round in shape, were observed (Supplementary Fig. 2 and 3). “Parking lot” paracrystalline inclusions were not observed.

### Genetic analysis

Genetic analysis was performed in all 11 patients (1 WES and 10 WGS). No variants that fulfilled the criteria to be likely pathogenic or pathogenic according to the American College of Medical Genetics and Genomics (ACMG) that could explain a metabolic disorder with an autosomal recessive inheritance were identified [22]. In one patient, a single heterozygous variant of uncertain significance (VUS) was identified in *ETFDH*.

**Fig. 4:**
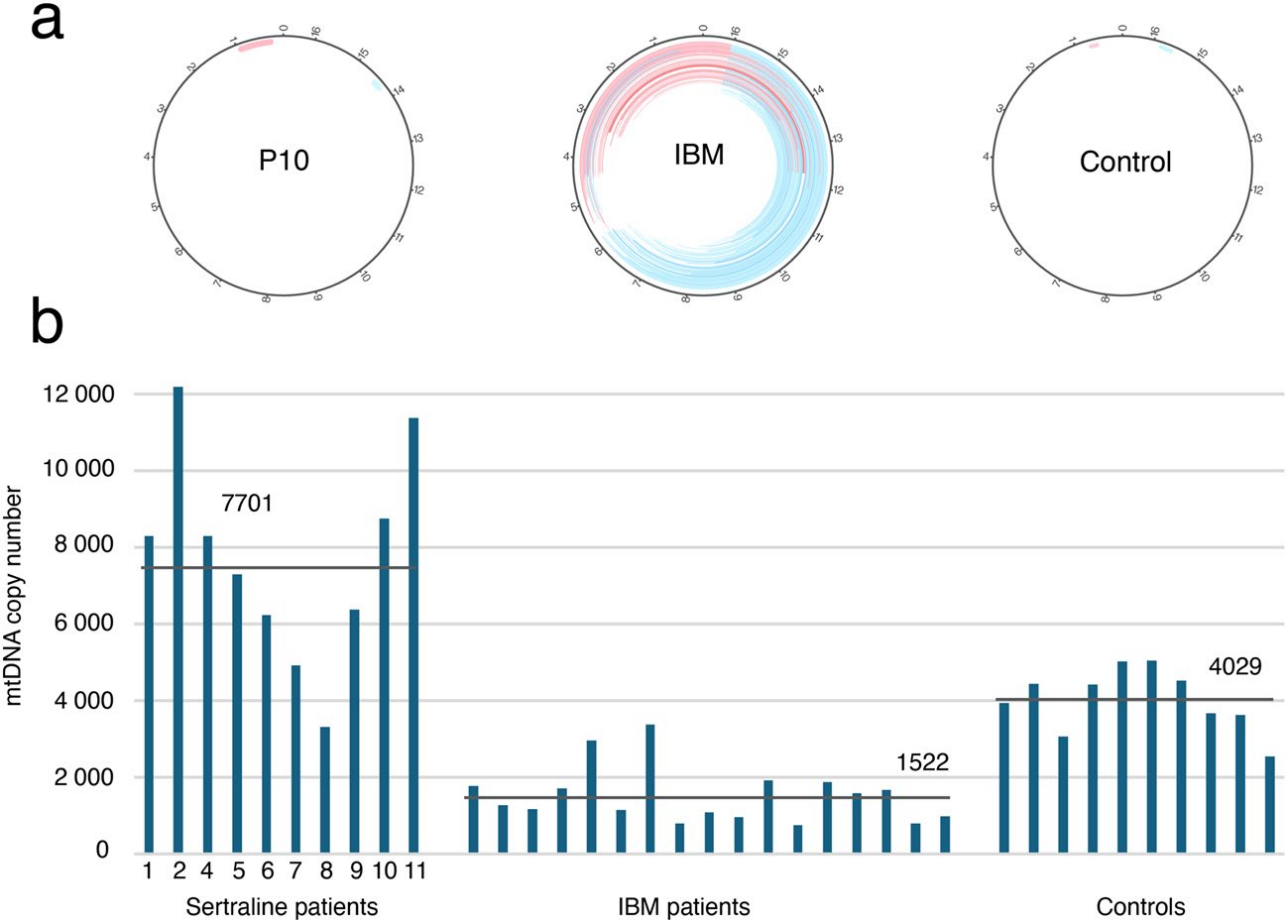
Investigation of mtDNA rearrangements and mtDNA copy number. **a** No increased number of mtDNA deletions or duplications were identified in patients with lipid storage myopathy associated with sertraline treatment. The circles illustrate mtDNA. The red (duplications) and blue (deletions) lines represent large-scale rearrangements of mtDNA. The intensity of each line corresponds to the number of specific deletions/duplications. For comparison, a typical example of patients with inclusion body myositis (IBM) with multiple deletions and duplications and a normal control is illustrated. **b** The mtDNA copy number were in general increased in the patients with lipid storage myopathy associated with sertraline treatment, which may reflect the increased number of mitochondria. For comparison, normal controls and a representative group of patients with IBM that frequently show reduced mtDNA copy numbers in spite of mitochondrial proliferation are illustrated

Detailed characterization of mtDNA was performed in 10 patients after WGS of skeletal muscle DNA, which showed a mean read depth of mtDNA of 157,500x. Bioinformatic analysis did not reveal any increase of large scale mtDNA deletions or duplications in any of the lipid storage myopathy cases compared to controls (Fig. 4a). The mtDNA copy number was in general higher in the patients with lipid storage myopathy with a mean of 7701 mtDNA copies per 1 nuclear DNA copy (range 3314–12,178) compared to controls muscle (mean 4029; range 2552–5054) (Fig. 4b).

### Proteomic profiling by TMT based LC-MS^3^ analysis

**Fig. 5:**
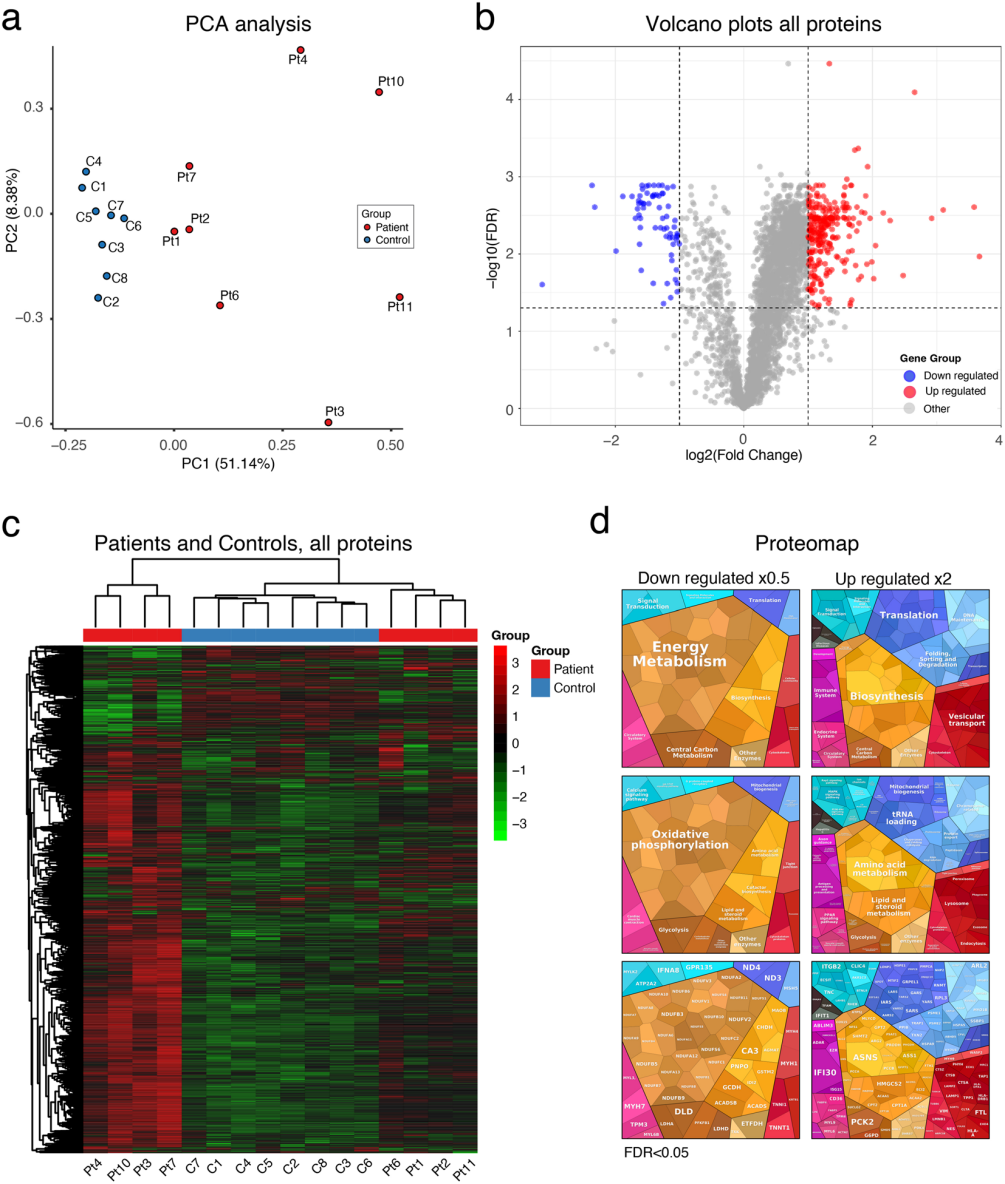
Basic proteomic data. **a** Principal component analysis (PCA) shows that the controls are clustered together and no apparent outliers. **b** Volcano plot including all approximately 3600 quantified proteins illustrating sertraline group versus controls. The proteins with an adjusted *p* value (false discovery rate, FDR) <0.05 and a fold change of <0.5 (log2FC < −1) (blue) or a fold change of >2 (log2FC > 1) (red) are indicated. **c** Heatmap displays the expression of differentially expressed proteins identified from the proteomics analysis. Each column corresponds to one sample (P, patient; C, control). Red indicates high expression level; green indicates low expression level. Hierarchical clustering shows the dendrogram based on the differences in protein profile for patients and controls, and shows difference in the clustering between patients and controls. **d** Proteomap shows that many significantly downregulated proteins with a fold change of less than 0.5 are involved in oxidative phosphorylation

On the background of lipid accumulation, mitochondrial proliferation, abnormal mitochondrial structure, apparently reduced activity of Complex II and IV as seen on enzyme histochemistry (COX and SDH), and accumulation of acylcarnitines of various lengths in blood, all pointing toward a mitochondrial dysfunction, we studied the expression of mitochondrial proteins in the proteome of the muscle-biopsy specimens. The analysis included eight patients and eight age-matched controls. The quantitative analysis was based on nanoscale liquid chromatography coupled to TMT based LC-MS^3^. From the basic analysis of the proteomic data, around 4,300 proteins were identified. Of these, 3,600 were identified in all samples and quantified. The principal component analysis (PCA) showed that the controls were distributed as a group separate from the patients (Fig. 5a). 1,926 proteins were significantly different (adjusted *p* value (FDR) < 0.05) between the control group and the sertraline group (Fig. 5b), the majority being upregulated in the sertraline group (Fig. 5b, c). The downregulated proteins were mainly associated with mitochondria, especially the respiratory chain in spite of the mitochondrial proliferation (Fig. 5d, the maps are generated at the Proteomap website (www. https://www.proteomaps.net/; [16]). In the heat map as well as in the PCA analysis based on the overall protein profiling the controls are more homogeneous than the patients. In the heat map the patients form two clusters, both separate from the control cluster. It is not clear from clinical or pathological features why the patients fall into two clusters.

**Fig. 6:**
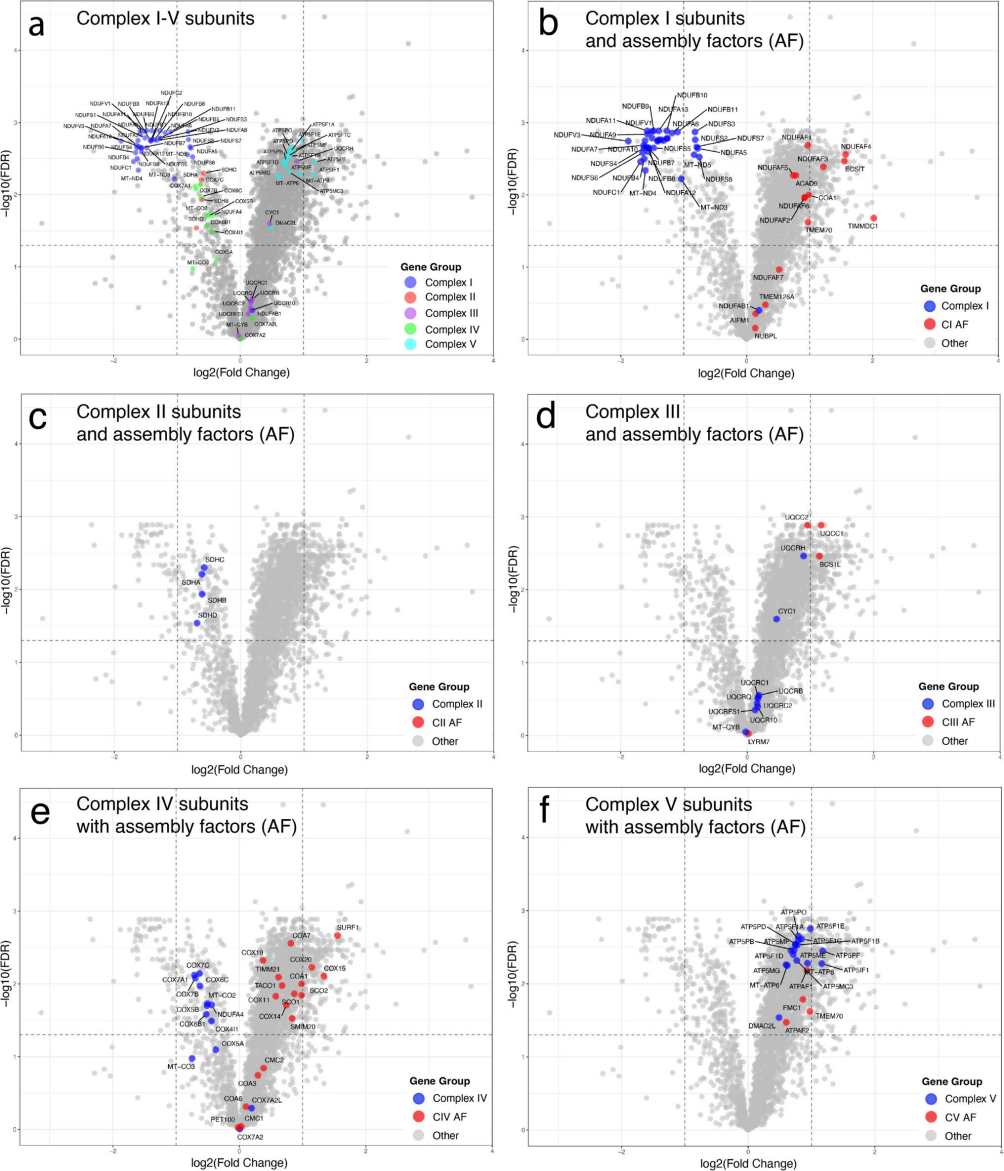
Volcano plots of subunits and assembly factors involved in Complex I–V in the respiratory chain (oxidative phosphorylation) **a** Volcano plots of subunits of Complex I–V showing downregulation of mainly Complex I but also of Complex II and IV, whereas Complex II and V are upregulated and possibly unchanged in relation to the increased number of mitochondria. **b–f** Volcano plats of the individual complexes I–V including also assembly factors. While the subunits of Complex I, II and IV are all statistically downregulated most assembly factors are up regulated and possibly unchanged in relation to the increased number of mitochondria

**Fig. 7:**
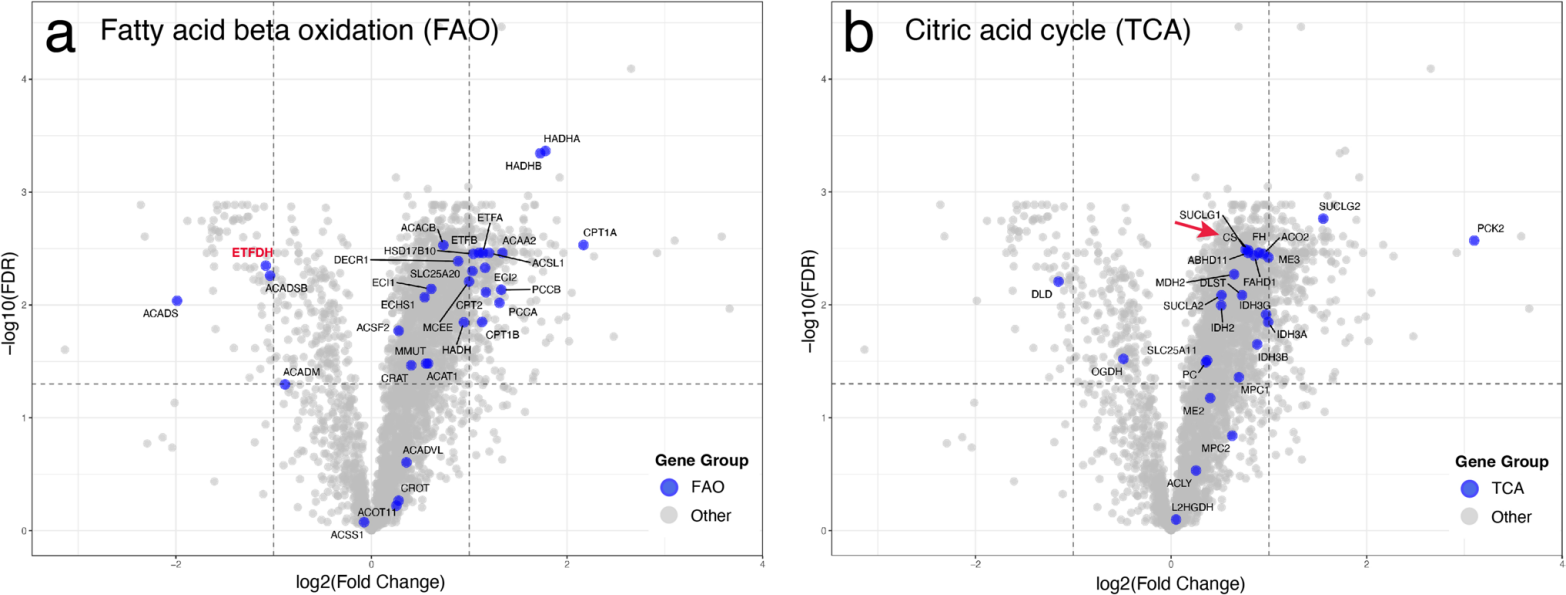
Volcano plots of proteins involved in fatty acid ß-oxidation (**a**) and citric acid cycle (**b**). Most of these enzymes appear to be up-regulated and possibly unchanged in relation to the increased number of mitochondria. One exception is *ETFDH* encoding the ETF-QO complex which is significantly downregulated more than twofold (red symbol in **a**). Citrate synthase (CF), a common biochemical marker for overall mitochondrial volume, is significantly upregulated in line with the increased number of mitochondria (red arrow). For explanation of gene symbols see Supplementary Table 5

To study the most important pathways involved in the fatty acid energy metabolism we analyzed the five individual complexes of the respiratory chain (CI-CV), the ß-oxidation (FAO) and the citric acid cycle (TCA-cycle) (Figs. 6, 7 and Supplementary Table 4). Analysis of the subunits of the five enzyme complexes of the respiratory chain showed that Complex I was markedly downregulated. Most of all significantly downregulated proteins with an average fold change of less than 0.5 were Complex I subunit proteins (NADH dehydrogenase) (Fig. 6a). The subunits of Complex II (succinate dehydrogenase) were all downregulated but with a lesser fold change than Complex I (Fig. 6a). The vast majority of Complex IV (cytochrome c oxidase) subunits were also downregulated (Fig. 6a). On the other hand, subunits of Complex III and V were generally unchanged or upregulated (Fig. 6a). Including assembly proteins in the analysis revealed that the assembly factors of Complex I were generally upregulated, which was different from the subunits (Fig. 6b). No assembly factors of Complex II were identified in all samples and therefore not quantified (Fig. 6c). In contrast, most of Complex III, IV and V assembly factors were upregulated (Fig. 6d–f).

For explanation of gene symbols see Supplementary Table 4.

The enzymes involved in FAO were in general upregulated (Fig. 7a). An interesting exception being ETF-coenzyme Q oxidoreductase (ETF:CQ) encoded by *ETFDH*, which was significantly downregulated with a fold change of less than 0.5.

Likewise, the enzymes of the TCA cycle were generally upregulated including citrate synthase (Fig. 7b). This upregulation probably reflects the increased number of mitochondria.

### Immunofluorescence analysis of the respiratory chain (MITIF)

**Fig. 8:**
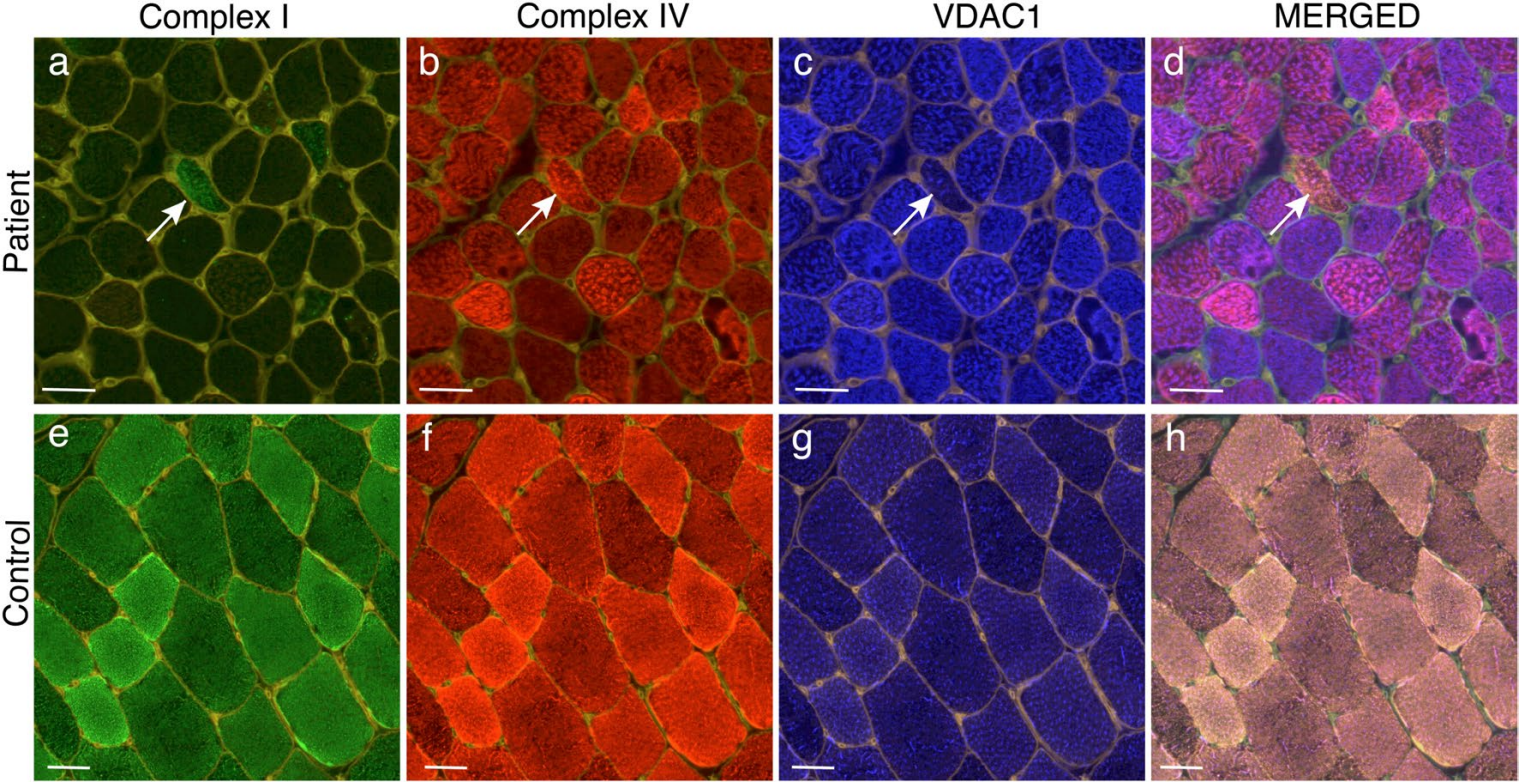
Quadruple immunofluorescence assay of Complex I and IV of the respiratory chain in Patient P10 (**a**–**d**) and a simultaneously stained control (**e**–**h**). In merged illustrations (**d** and **h**) yellow fibers are normal, red fibers are Complex I deficient, green fibers are Complex IV deficient and blue fibers are deficient of both Complex I and IV. There is profound deficiency of Complex I (**a**) and partial deficiency of Complex IV (**b**) in P10. Only occasional fibers show preserved Complex I and IV (arrow). The increased intensity of VDAC1 staining in many muscle fibers in P10 indicates increased number of mitochondria. Bars = 50 µm

**Fig. 9:**
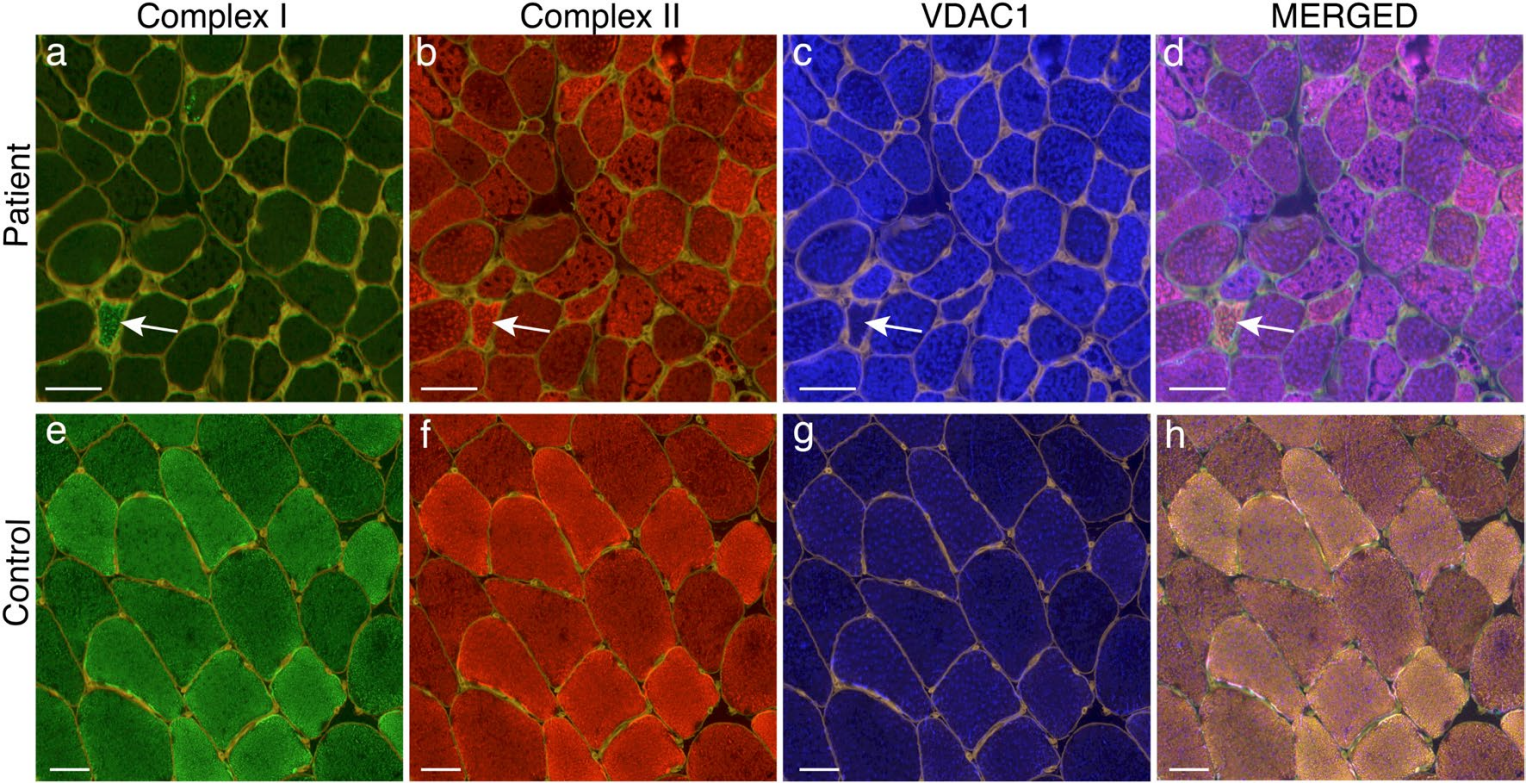
Quadruple immunofluorescence assay of Complex I and II of the respiratory chain in Patient P10 (**a**–**d**) and a simultaneously stained control (**e**–**h**). In merged illustrations (**d** and **h**) yellow fibers are normal, red fibers are Complex I deficient, green fibers are Complex II deficient and blue fibers are deficient of both Complex I and IV. There is profound deficiency of Complex I (**a**) and partial deficiency of Complex II (**b**) in P10. Only occasional fibers show preserved Complex I and IV (arrow). The increased intensity of VDAC1 staining in many muscle fibers in P10 indicates increased number of mitochondria. Bars = 50 µm

The proteomic results indicated downregulation mainly of respiratory chain subunits of Complex I and to some extent of Complex II and IV. We performed immunofluorescence analysis to study the cellular distribution of these complexes taking the mitochondrial proliferation in individual fibers into account by simultaneous staining of a mitochondrial membrane protein (porin, VDAC1). There was a profound deficiency of Complex I (NDUFB8) in most of the patients and only occasional muscle fibers expressed normal levels in relation to the increased number of mitochondria (Figs. 8 and 9, Table 2, Supplementary Fig. 4). For Complex II (SDHB) and IV (MT-CO1), the deficiency was less pronounced compared to the Complex I deficiency. The level of complex II and IV varied between different adjacent muscle fibers in a mosaic pattern (Figs. 8 and 9, Table 2). There was evidence of an increased number of mitochondria in all patients (Figs. 8 and 9, Table 2).

### Western blot analysis

Western blot analysis was performed with the same antibodies as the immunofluorescence assay in addition to Complex III (UQCRC2) and Complex V (ATPB). The overall pattern showed a reduced amount of Complex I, II and IV but no reduction of Complex III and V (Table 2 and Supplementary Fig. 5).

## Discussion

In this study we describe 11 patients with lipid storage myopathy associated with sertraline treatment. We demonstrate a profound and consistent deficiency of Complex I in the respiratory chain together with proliferation of ultrastructurally abnormal mitochondria. These results confirm the previously suspected association between sertraline treatment and lipid storage myopathy and provide morphological and biochemical characteristics in this disease. Our findings also indicate that acquired lipid storage myopathy associated with sertraline treatment is by far the most common form of lipid storage myopathy in western Sweden, which is in accordance with the study from the southeastern part of Sweden by Sunebo et al. [26].

Lipid storage myopathies are traditionally defined as a group of genetic metabolic disorders showing pathological accumulation of lipid droplets in the muscle fibers [2, 6]. They are usually associated with defects of transport and oxidation of exogenous fatty acids or endogenous triglyceride catabolism [6]. Diagnosis involves investigation of acylcarnitines in blood and analysis of excreted organic acids in urine and identification of pathogenic variants in specific genes [2, 24]. One of these disorders, MADD or glutaric aciduria type II is usually caused by biallelic pathogenic variants in the gene *ETFDH* encoding ETF-CQ or genes encoding electron-transfer flavoproteins (*ETFA*, *ETFB*) [12, 20, 27]. MADD type III (late onset) may present with muscle weakness, fatigue and lipid storage myopathy [27]. There are also other genetic causes of muscle weakness with MADD-like acylcarnitine profile such as biallelic pathogenic variants in genes-encoding enzymes involved in riboflavin metabolism (*FLAD1*, *SLC25A32*, *SLC52A1*, *SLC52A2*, *SLC52A3*) [19] and pathogenic variants in mtDNA [23].

Sertraline is a selective serotonin uptake inhibitor widely used as an antidepressant. It is well-known that side effects include myalgia, muscle weakness and rhabdomyolysis [7, 11, 18, 25]. Recently, Sunebo et al. [26], in a systematic retrospective single center study, identified nine adult patients with lipid storage myopathy and a MADD-like acylcarnitine profile. Two patients carried apparently pathogenic biallelic variants in *ETFDH* whereas seven patients were not identified with a probable genetic cause. All these seven patients were treated with sertraline at the onset of symptoms, indicating that sertraline in some patients may cause a lipid storage myopathy with a MADD-like acylcarnitine profile. In a case report, one patient with similar clinical phenotype, muscle biopsy showed lipid storage and mitochondrial changes on electron microscopy [15]. In a study from Australia, ten of 18 adult patients diagnosed with glutaric aciduria type II, based on the acylcarnitine profile but without a genetic diagnosis, were taking sertraline [9]. It was not reported whether these patients had a lipid storage myopathy, but the majority had muscle symptoms such as myalgia, fatigue and myopathy.

We have investigated muscle-biopsy specimens from 11 patients with lipid storage myopathy associated with sertraline treatment. First, we demonstrate abnormal and proliferating muscle mitochondria based on muscle enzyme histochemistry, electron microscopy and increased copy number of mtDNA. By proteomic analysis applying quantitative mass spectrometry we identified a profound deficiency of subunits of the respiratory chain Complex I, and to some extent Complex II and IV. By a quadruple immunofluorescence analysis, the results from proteomic analysis were verified and we demonstrated mitochondrial proliferation and deficiency of Complex I, II and IV at the cellular level. These results were also supported by western blot analysis. The protein components of Complex III and V were not affected. The clinical, biochemical (acylcarnitine profile), histopathological, electron microscopical and proteomic findings show striking similarities within the group of patients indicating a common pathogenesis which apparently includes treatment with sertraline. Our proteomic results indicate upregulation of several metabolic pathways of fatty acid transport and oxidation in line with the findings of markedly increased numbers of mitochondria in the muscle tissue. The overall loss of Complex I subunits is in this respect remarkable and indicates that this part of the respiratory chain is severely affected in lipid storage myopathy associated with sertraline treatment. Although MADD-like acylcarnitine profile and lipid storage myopathy may occur secondary to respiratory chain deficiency it is usually not a characteristic finding. Therefore, loss of ETF:QO (encoded by *ETFDH*) from the mitochondria as revealed by the proteomic analysis may be part of the explanation for the MADD-like changes in addition to the profound deficiency of Complex I.

Sertraline is internationally one of the most prescribed drugs. The estimated number of patients in the United States 2022 were 8.4 millions (ClinCalc DrugStats Database version 2024.08 https://clincalc.com/DrugStats/). Due to the high usage, also rare side effects have the potential to affect many individuals. We believe the number of undiagnosed and clinically affected cases may be large and clinicians should therefore be aware of the adverse effects on mitochondrial function of sertraline. We did not observe patients with a presumably acquired lipid storage myopathy who were treated with other antidepressant drugs. Still, an increase of short-chain acylcarnitines has been seen in blood during treatment with citalopram and escitalopram, which are selective serotonin reuptake inhibitors similar to sertraline [17].

Since lipid storage myopathy appears to be a rare event in patients on sertraline treatment there may be trigger factors and/or genetic susceptibility involved. Sertraline is metabolized by CYP enzymes and pharmocogenetic studies suggest that CYP2C19 is the major metabolic enzyme [5]. Since some variants in the *CYP2C19* gene called *alleles, are reported to affect the enzyme activity, we analyzed the presence of these variants in our patients. The results are shown in Supplementary material Table 6. From this analysis we could not see any clear association between analyzed *alleles and disease. However, to be able to draw any general conclusions regarding association with lipid storage myopathy a much larger cohort of patients is warranted. It has been suggested that heterozygous pathogenic variants in genes that are associated with MADD may develop glutaric aciduria type II [9]. However, we did not find any pathogenic variants in *ETFDH*, *ETFA* or *ETFB* in any of our 11 patients with lipid storage myopathy associated with sertraline treatment, which is line with previous studies [15, 26].

Our results show that lipid storage myopathy associated with sertraline treatment is a mitochondrial disorder with respiratory chain deficiency and is an important differential diagnosis with characteristic features. Clinicians should be aware of the adverse effects on mitochondrial function of sertraline causing muscle weakness and a MADD-like acylcarnitine profile.

## Supplementary Information

Below is the link to the electronic supplementary material.

Supplementary file1 (PDF 32632 KB)

## Data Availability

No datasets were generated or analysed during the current study.
