# Supplementary material for: Lipid storage myopathy associated with sertraline treatment is an acquired mitochondrial disorder with respiratory chain deficiency
Source: Acta Neuropathol. 2024 Nov 26;148(1):73. doi: 10.1007/s00401-024-02830-x (PMC11588938; doi:10.1007/s00401-024-02830-x)
Supplement: Supplementary file 1 — Supplementary file1 (PDF 32632 KB) [file 401_2024_2830_MOESM1_ESM.pdf]

+

Supplementary information

Lipid storage myopathy associated with sertraline treatment is an acquired mitochondrial disorder with respiratory chain deficiency

Carola Hedberg-Oldfors[1], Ulrika Lindgren[1,2], Kittichate Visuttijai[1], Yan Shen[1], Andreea Ilinca[3,4], Sara Nordström[2], Christopher Lindberg[2] and Anders Oldfors[1]

[1] Department of Laboratory Medicine, University of Gothenburg, Gothenburg, Sweden

[2] Neuromuscular Centre, Department of Neurology, Sahlgrenska University Hospital, Gothenburg, Sweden

[3] Department of Neurology, Skåne University Hospital, Division of Neurology, Lund, Sweden

[4] Department for Clinical Sciences, Lund University, Lund, Sweden.

Corresponding author: Anders Oldfors; Department of Laboratory Medicine, University of Gothenburg, Gothenburg, Sweden

Address: Sahlgrenska University Hospital, Gula stråket 8, 413 45 Gothenburg, Sweden, phone number +46 31 342 2084, e-mail: anders.oldfors@gu.se

Material and Methods

Supplementary Table 1. Detailed laboratory findings in all patients

Patient	Acylcarnitines, free carnitine (C0)	U-organic acids	Lactic acid (serum) (0,5-1,7 mmol/L)
1	Increased C12, C14, C16, C18, and C18:1, slightly reduced C0 (20 days after biopsy)	No pathological excretion	**1.9** (3 month after biopsy)<br>1.4 (14 month after biopsy)<br>1.5 (16 month after biopsy)<br>**2.0** (3 years after biopsy)<br>**2.2** (7 years after biopsy
2	Increased C6, C8, C10, C12, C14, C16, C14:1, C18:1 and C18 (25 days after biopsy)		**3.2** (3 month after biopsy)
3	Carnitine deficiency with low concentration of C0 and total carnitines (same day as biopsy)		
4	Increased C4, C6, C10, C12, C14, C16, C14:1, C18:1 and C18, slightly reduced C0 (30 days after biopsy)	Increased excretion of ethylmalonic acid (EMA) and 2-hydroxyglutaric acid (2-HGA) (35 days after biopsy)	1.3 (4 month after biopsy)
5	Increased C6, C8, C10 and C12 (1 month after biopsy)<br>Increased C4, C6, C8, C10, C12, C14 and C14:1 (2 month after biopsy)		
6	Increased C4, C6, C10, C12, C14, C16, C14:1, C18:1 and C18 (7 days before biopsy)	Increased excretion of EMA and 2-HGA, adipic acids and isovalerylglycine (7 days before biopsy)	
7	Carnitine deficiency with low concentration of C0 and total carnitines (3 days after biopsy)	No patological excretion (23 days after biopsy)	
8	Increased C6, C8, C10, C12, C14, C16, C14:1, C14:2, C18:1, C18 and C5DC (8 month after biopsy)		
9	Increased C12 (1.5 month after biopsy), Increased C12, C14 and C14:1 (6 month after biopsy)	No pathological excretion (6 month after biopsy)	1.2 (6 month after biopsy)
10	Increased C6, C8, C10, C12, C14, C16, C14:1, C18:1 (4 month after biopsy)	No pathological excretion (4 month after biopsy)	1.2 (4 month after biopsy)
11	Increased C4, C5, C6, C8, C10, C12, C14, C16, C14:1, C14:2, C18:1, C18 and C5DC (1.5 month after biopsy)	No pathological excretion (1,5 month after biopsy)	**3.1** (1.5 month after biopsy)

Supplementary Table 2. A summary of controls used in this study

Method	Muscle biopsy findings	Age at biopys (Y)	Sex
Western blot			
DC1	Muscle biopsy with lipid storage (MADD, *ETFDH*)	25	M
DC2	Muscle biopsy with lipid storage (Neutral lipid storage disease with myopathy, *PNPLA2*)	43	F
C1	Normal muscle biopsy	22	M
C2	Normal muscle biopsy	45	F
C3	Normal muscle biopsy	58	F
Proteomic profiling			
C1	Normal muscle biopsy	52	M
C2	Same as C1 in western blot	22	M
C3	Normal muscle biopsy	61	M
C4	Normal muscle biopsy	48	M
C5	Same as C2 in western blot	45	F
C6	Normal muscle biopsy	43	F
C7	Normal muscle biopsy	26	F
C8	Same as C3 in western blot	58	F

Y, year; F, female; M, Male; DC, disease control; C, control (from individuals who had been investigated for a possible muscle disorder, but in whom investigations excluded muscle disease)

Immunofluorescence analysis of respiratory chain Complexes I, II and IV

Cryosections from each patient were divided into two separate staining sets: one set included complexes I, II, VDAC1, and perlecan and the other set included complexes I, IV, VDAC1, and perlecan. The sections were fixed in 4% formaldehyde in 0.1 M phosphate buffer for 10 minutes at 4°C, then rinsed with water three times and washed in Tris-buffered saline with Tween 20 (TBS-T) for 10 minutes. Permeabilisation was carried out through a graded methanol series (70% 2 minutes, 95% 2 minutes, 100% 10 minutes, 95% 2 minutes, and 70% 2 minutes), followed by another wash in TBS-T for 5 minutes. The sections were subsequently processed using a Dako Autostainer, where the sections were blocked with 10% normal goat serum before incubating with the primary antibodies and the corresponding fluorochrome-conjugated secondary antibodies (Supplementary Table 2).

All slides were scanned using a Hamamatsu S60 digital scanner with fluorescence equipment, including a DAPI/FITC/TRITC/Cy5 quad-band filter set (Semrock, New York). Results from studies using this assay on biochemically and genetically verified cases with isolated Complex I and IV deficiency and well characterized and genetically defined patients with combined complex I and IV deficiency are shown in Supplementary Fig 1.

Supplementary Table 3. Antibodies for immunofluorescence and western blot analyses

**Antigen** (Isotype/Conjugation)	**Host**	**Dilution**			**Manufacturer** (Catalogue Number)
		MIFA	MITIF	WB	
		Primary antibodies			
**NDUFB8 (Complex I)** (IgG1)	Mouse		1:200	1:1000	Abcam (ab110242)
**SDHB (Complex II)** (IgG2a)	Mouse		1:200	1:1000	Abcam (ab14714)
**UQCRC2 (Complex III)** (IgG1)	Mouse			1:2000	Abcam (ab14745)
**MTCO1 (Complex IV)** (IgG2a)	Mouse		1:200	1:1000	Abcam (ab14705)
**ATPB (Complex V)** (IgG1)	Mouse			1:2000	Abcam (ab14730)
**VDAC1 (Porin)** (IgG2b)	Mouse		1:1000		Abcam (ab14734)
**VDAC1** (IgG)	Rabbit			1:1000	Cell Signaling Technology (4661)
**MYH7 (MyHC I)** (IgG2b)	Mouse	1:50			DSHB (BA-D5)
**MYH2 (MyHC IIa)** (IgG1)	Mouse	1:50			DSHB (SC-71)
**MYH1 (MyHC IIx)** (IgM)	Mouse	1:10			DSHB (6H1)
**Heparan Sulfate Proteoglycan (Perlecan)** (IgG2aκ)	Rat	1:200	1:1000		Merck (MAB1948P)
		Secondary antibodies			
**Mouse IgG** (**HRP**)	Goat			1:1000	ThermoFisher Scientific (32430)
**Rabbit IgG** (**HRP**)	Goat			1:1000	ThermoFisher Scientific (32460)
**Mouse IgG1** (Alexa Fluor 488)	Goat	1:200	1:200		ThermoFisher Scientific (A-21121)
**Mouse IgM (H)** (Alexa Fluor 647)	Goat	1:200			ThermoFisher Scientific (A-21238)
**Mouse IgG2a** (Alexa Fluor 647)	Goat		1:100		ThermoFisher Scientific (A-21241)
**Mouse IgG2b** (Brilliant Violet 421)	Goat	1:200	1:100		Jackson ImmunoResearch (115-675-207)
**Rat IgG (H+L)** (Alexa Fluor 568)	Goat	1:200	1:250		ThermoFisher Scientific (A-11077)

HRP: Horseradish peroxidase

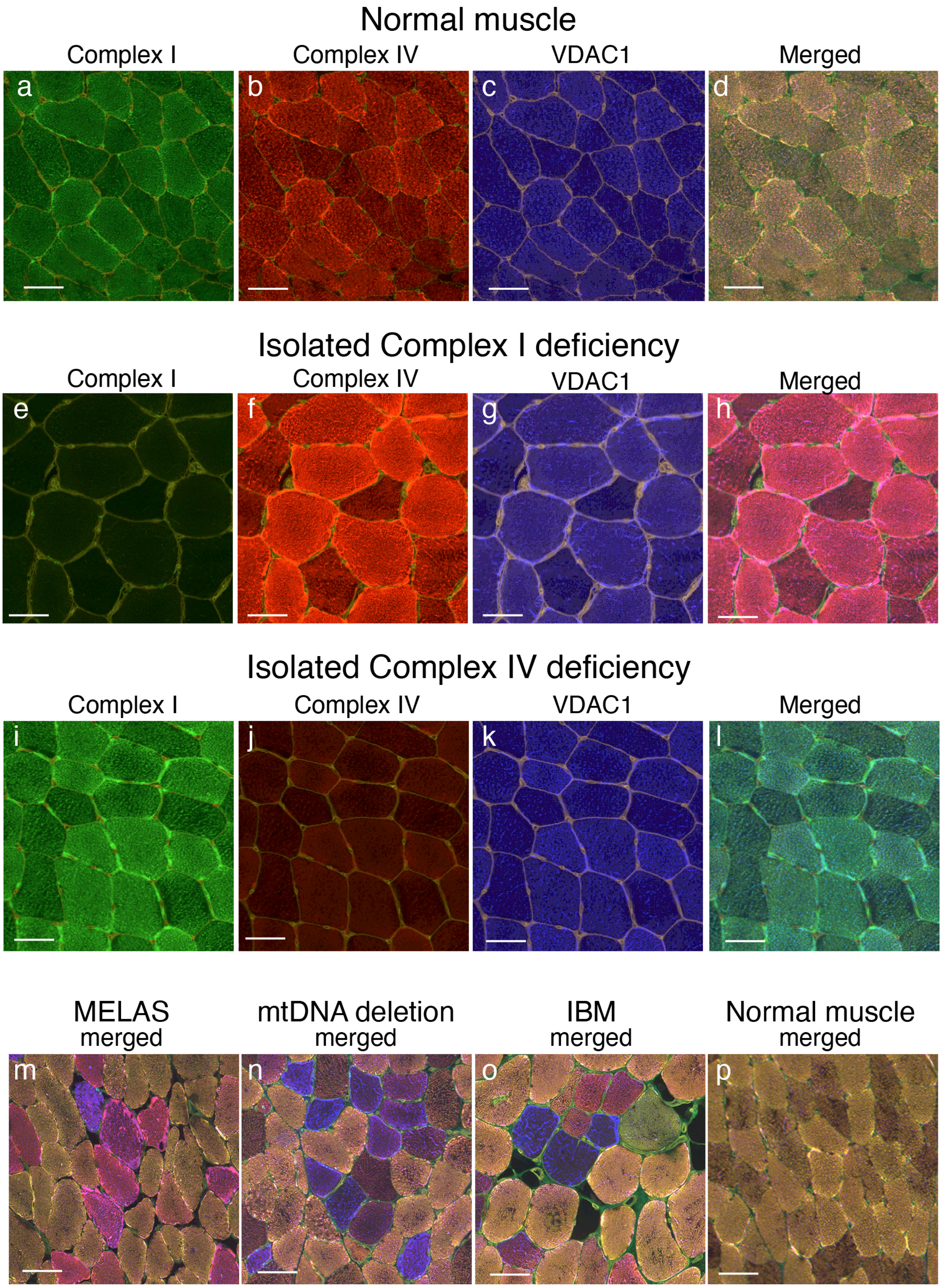

Supplementary Fig 1. Quadruple immunofluorescence assay applied on well characterized diseases and normal muscle. In merged illustrations yellow fibers are normal, red fibers are Complex I deficient, green fibers are Complex IV deficient and blue fibers are deficient of both Complex I and IV.

a-d) Normal muscle. e-h) Patient with isolated Complex I deficiency (Darin et al. Eur J Neurol. 2017;24:587-93). i-l) Patient with isolated Complex IV analysis (Roos et al. Eur J Hum Genet. 2019;27:331-5). m) mtDNA variant m.3243A>G associated with mitochondrial encephalomyopathy lactic acidosis and stroke-like periods (MELAS). n) Single large scale mtDNA deletion o) Multiple large and small deletions in inclusion body myositis (IBM). p) Normal control. Bars = 50 µm.

Proteomic Liquid Chromatography-Mass Spectrometry (LC-MS^3) analysis

Sample preparation

Proteins were extracted in 2% sodium dodecyl sulfate and 100 mM triethylammonium bicarbonate (TEAB) using a Covaris ML230 ultrasonicator. Protein concentrations were determined using Pierce BCA Protein Assay Kit (Thermo Scientific) on a SpectraMax iD3 microplate reader (Molecular Devices). Samples (40 µg) were processed using a modified SP3 method. Samples were reduced in 10mM dithiothreitol at 56°C for 30min and alkylated in 20mM iodoacetamide at room temperature for 30min. Washed hydrophobic and hydrophilic Sera-Mag™ SpeedBeads (Carboxylate-Modified, by Cytiva) were added to the samples with a bead to protein ratio of 18:1. Proteins were precipitated on the beads by ethanol (final concentration 70%), washed twice with 70% ethanol, followed by wash with 100% acetonitrile, dried at room temperature. Beads were resuspended in 50mM TEAB and proteins were digested with Trypsin/Lys-C mix (1:25, Promega) for two hours, followed by trypsin (1:50, Promega) overnight. The magnetic beads were removed, and samples were labelled using TMTpro 18-plex isobaric mass tagging reagents (Ref#: A52045; Lot#: XI346567 and XJ346678, Thermo Fisher Scientific). The labelled samples were pooled and purified using HiPPR Detergent Removal Resin and Pierce peptide desalting spin columns (both Thermo Scientific), according to the manufacturer´s instructions. The TMT-set was fractionated by basic reversed-phase chromatography on a Dionex Ultimate 3000 UPLC system (Thermo Fisher Scientific). Peptide separations were performed using a reversed-phase XBridge BEH C18 column (3.5 µm, 2.1x250 mm, Waters Corporation) and a stepped gradient from 3% to 80% solvent B over 90 min at a flow of 200 µL/min. Solvent A was 25mM ammonia and solvent B was 85% acetonitrile. Fractions (96) were collected from 20-74 minutes and combined into 36 final fractions. Fractions were evaporated and reconstituted in 3% acetonitrile, 0.1% trifluoroacetic and 0.015% dodecyl-β-D-maltoside for LC-MS^3 analysis.

LC-MS^3 analysis

The fractions were analysed on an Orbitrap Lumos™ Tribrid™ mass spectrometer equipped with a FAIMS Pro ion mobility system and interfaced with an Easy-nLC1200 liquid chromatography system (all Thermo Fisher Scientific). Peptides were trapped on an Acclaim Pepmap 100 C18 trap column (100 µm x 2 cm, particle size 5 µm, Thermo Fisher Scientific) and separated on an in-house packed analytical column (35 cm x 75 µm, particle size 3 µm, Reprosil-Pur C18, Dr. Maisch) using a stepped gradient from 5% to 35% B over 77 min at a flow of 300 nL/min (Solvent A 0.2% formic acid, solvent B 80% ACN and 0.2% formic acid). FAIMS Pro was alternating between the compensation voltages (CV) of -50 and -70, and the same data-dependent settings were used at both CVs. The precursor ion mass spectra were acquired at a resolution of 120 000 and an m/z range of 375-1375. Using a cycle time of 1.5 seconds the most abundant precursors with charges 2–7 were isolated with an m/z window of 0.7 and fragmented by collision-induced dissociation (CID) at 35%. Fragment spectra were recorded in the ion trap at a Rapid scan rate. Dynamic exclusion was set to 60 sec. The ten most abundant MS2 fragment ions were isolated using multi-notch isolation for further MS^3 fragmentation. MS^3 fragmentation was performed using higher-energy collision dissociation (HCD) at 55% and the MS^3 spectra were recorded in the Orbitrap at 50 000 resolution and an m/z range of 100–500.

Proteomic data analysis

Raw files were processed and analyzed with Proteome Discoverer (ver 3.0, Thermo Fisher Scientific) The data was matched against *Human* SwissProt database (20597 entries, February 2024) using Sequest as a search engine

with a precursor tolerance of 5 ppm and a fragment ion tolerance of 0.6 Da. Tryptic peptides were accepted with 1 missed cleavage. Methionine oxidation and acetylation on protein N-termini was set as variable modifications and cysteine carbamidomethylation, TMTpro on lysine and peptide N-termini were set as fixed modifications. Percolator was used for PSM validation with a strict FDR threshold of 1%. For quantification TMT reporter ions were identified in the MS^3 HCD spectra with 3 mmu mass tolerance and the TMT reporter intensity values for each sample were normalized on the total peptide amount. The SPS threshold was set to 65%, a Sequest HT threshold score of 2 was chosen. Only unique peptides were used for relative quantification and proteins were required to pass a protein FDR of 1%.

Quantitative LC-MS^3 proteomic analysis and illustration by volcano plots

For quantitative LC-MS^3 proteomic analysis and illustration by volcano plots of the respiratory chain Complex I-V, the beta-oxidation of fatty acids (FAO) and the citric acid cycle (TCA), genes were selected based on "MitoCarta3.0: An Inventory of Mammalian Mitochondrial Proteins and Pathways" (https://www.broadinstitute.org/mitocarta/mitocarta30-inventory-mammalian-mitochondrial-proteins-and-pathways).

Complex I

CI subunits

MT-ND1, MT-ND2, MT-ND3, MT-ND4, MT-ND4L, MT-ND5, MT-ND6, NDUFA1, NDUFA10, NDUFA11, NDUFA12, NDUFA13, NDUFA2, NDUFA3, NDUFA5, NDUFA6, NDUFA7, NDUFA8, NDUFA9, NDUFAB1, NDUFB1, NDUFB10, NDUFB11, NDUFB2, NDUFB3, NDUFB4, NDUFB5, NDUFB6, NDUFB7, NDUFB8, NDUFB9, NDUFC1, NDUFC2, NDUFS1, NDUFS2, NDUFS3, NDUFS4, NDUFS5, NDUFS6, NDUFS7, NDUFS8, NDUFV1, NDUFV2, NDUFV3

CI assembly factors

ACAD9, AIFM1, COA1, DMAC1, DMAC2, ECSIT, FOXRED1, LYRM2, NDUFAF1, NDUFAF2, NDUFAF3, NDUFAF4, NDUFAF5, NDUFAF6, NDUFAF7, NDUFAF8, NUBPL, TIMMDC1, TMEM126A, TMEM126B, TMEM186, TMEM70

Complex II

CII subunits

SDHA, SDHB, SDHC, SDHD

CII assembly factors

SDHAF1, SDHAF2, SDHAF4, SDHAF3

Complex III

CIII subunits

CYC1, MT-CYB, UQCR10, UQCR11, UQCRB, UQCRC1, UQCRC2, UQCRFS1, UQCRH, UQCRQ

CIII assembly factors

BCS1L, LYRM7, TTC19, UQCC1, UQCC2, UQCC3

Complex IV

CIV subunits

COX4I1, COX4I2, COX5A, COX5B, COX6A1, COX6A2, COX6B1, COX6B2, COX6C, COX7A1, COX7A2, COX7A2L, COX7B, COX7B2, COX7C, COX8A, COX8C, MT-CO1, MT-CO2, MT-CO3, NDUFA4

CIV assembly factors

CEP89, CMC1, CMC2, COA1, COA3, COA4, COA5, COA6, COA7, COA8, COX10, COX11, COX14, COX15, COX16, COX17, COX18, COX19, COX20, HIGD1A, PET100, PET117, PNKD, SCO1, SCO2, SMIM20, SURF1, TACO1, TIMM21, TMEM177

Complex V

CV subunits

ATP5F1A, ATP5F1B, ATP5F1C, ATP5F1D, ATP5F1E, ATP5IF1, ATP5MC1, ATP5MC2, ATP5MC3, ATP5MD, ATP5ME, ATP5MF, ATP5MG, ATP5MPL, ATP5PB, ATP5PD, ATP5PF, ATP5PO, DMAC2L, MT-ATP6, MT-ATP8

CV assembly factors

ATPAF1, ATPAF2, ATPSCKMT, FMC1, TMEM70

FAO - Fatty Acid Oxidation

DECR1, HSD17B10, ACAA2, ACAT1, ACACB, ACSS1, ACOT11, AMACR, CRAT, CPT1A, CPT1B, CPT2, ETFA, ETFB, ETFDH, ECI1, ECI2, ECHS1, HADH, ACSL1, ACSF2, ACADM, MCEE, MMUT, SLC25A20, CROT, PCCA, PCCB, ACADS, ACADSB, HADHA, HADHB, ACADVL

TCA - citric acid cycle

OGDH, ACO2, FAHD1, ACLY, CS, D2HGDH, DLD, DLST, FH, IDH3A, IDH3B, IDH3G, IDH2, L2HGDH, MDH2, SLC25A11, MPC1, MPC2, ME2, ME3, PCK2, PC, ABHD11, SUCLA2, SUCLG1, SUCLG2, SLC25A1

Results

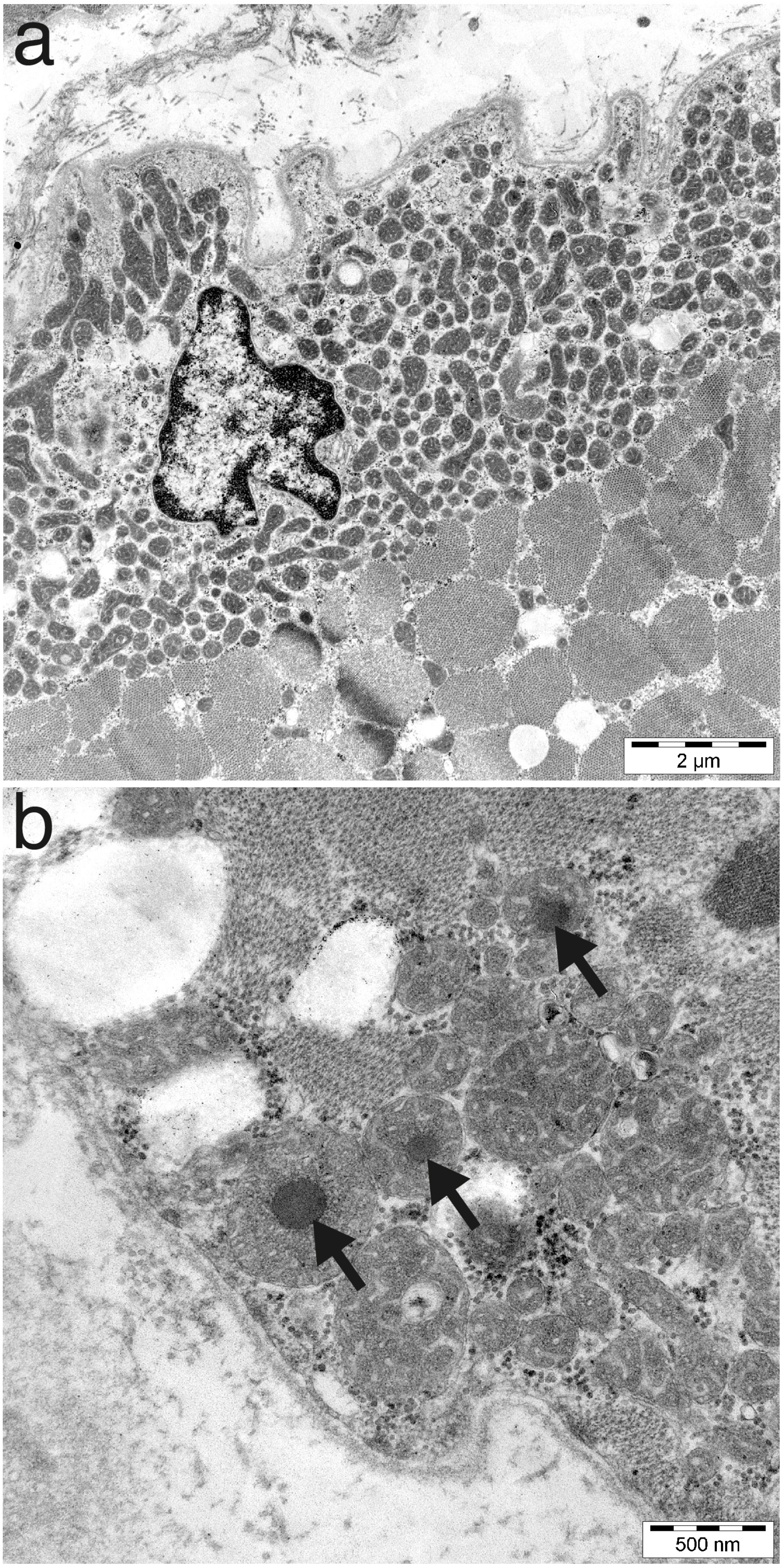

Supplementary Fig 2. Electron micrograph of muscle tissue in one patient (P1). a) Subsarcolemmal accumulation of pleomorphic mitochondria with dens matrix causing an overall dark appearance of the mitochondria. b) Some of the mitochondria show dark, round inclusions (arrows).

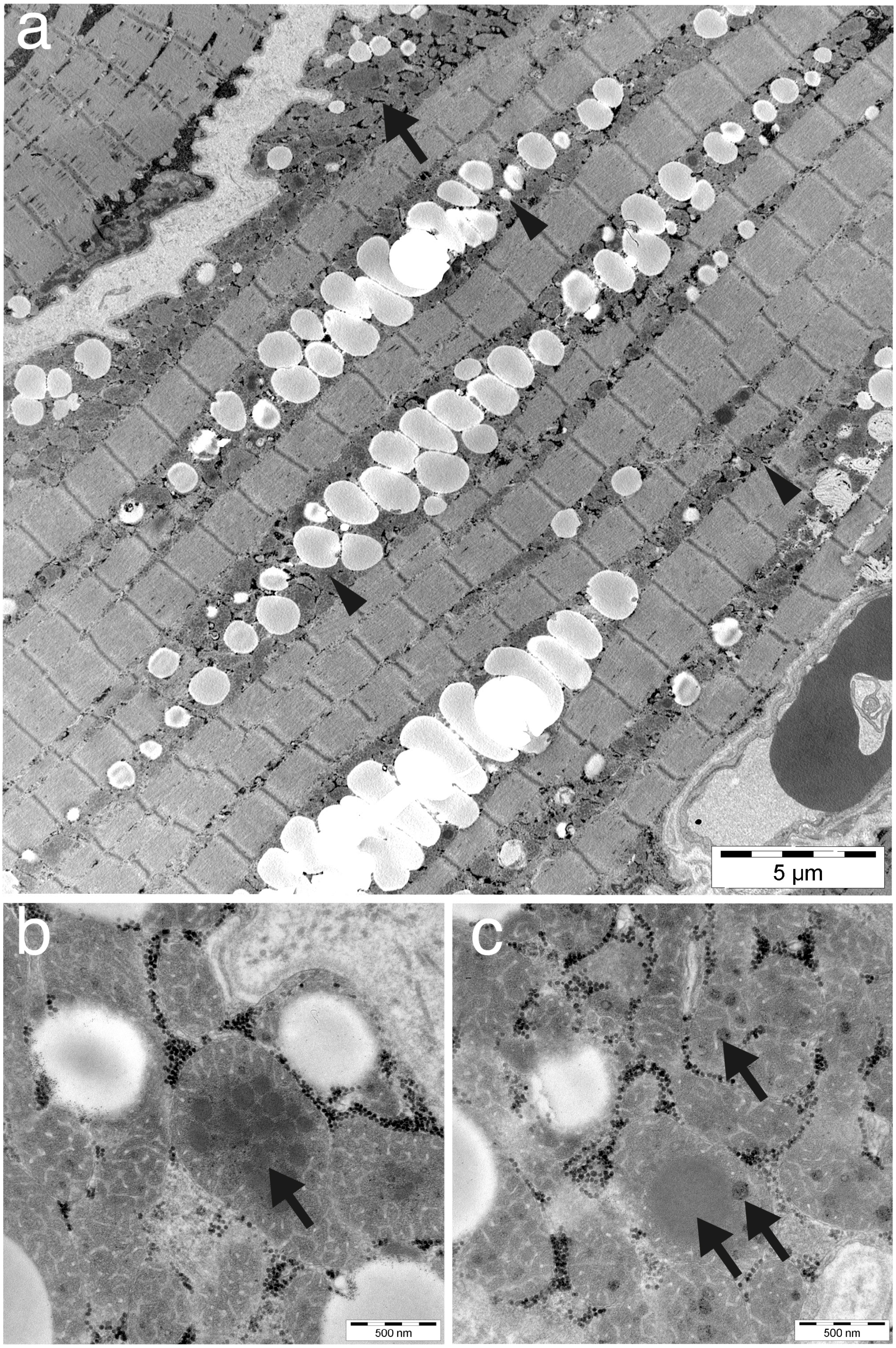

Supplementary Fig 3. Electron micrograph of muscle tissue in one patient (P2). a) The accumulation of mitochondria with electron-dense matrix causing an overall dark appearance. They are located in the sub-sarcolemmal region (arrow) as well as in the intermyofibrillar compartment associated with lipid storage (arrow heads). b and c) Many of the mitochondria show electron dense, round inclusions of various sizes (arrows).

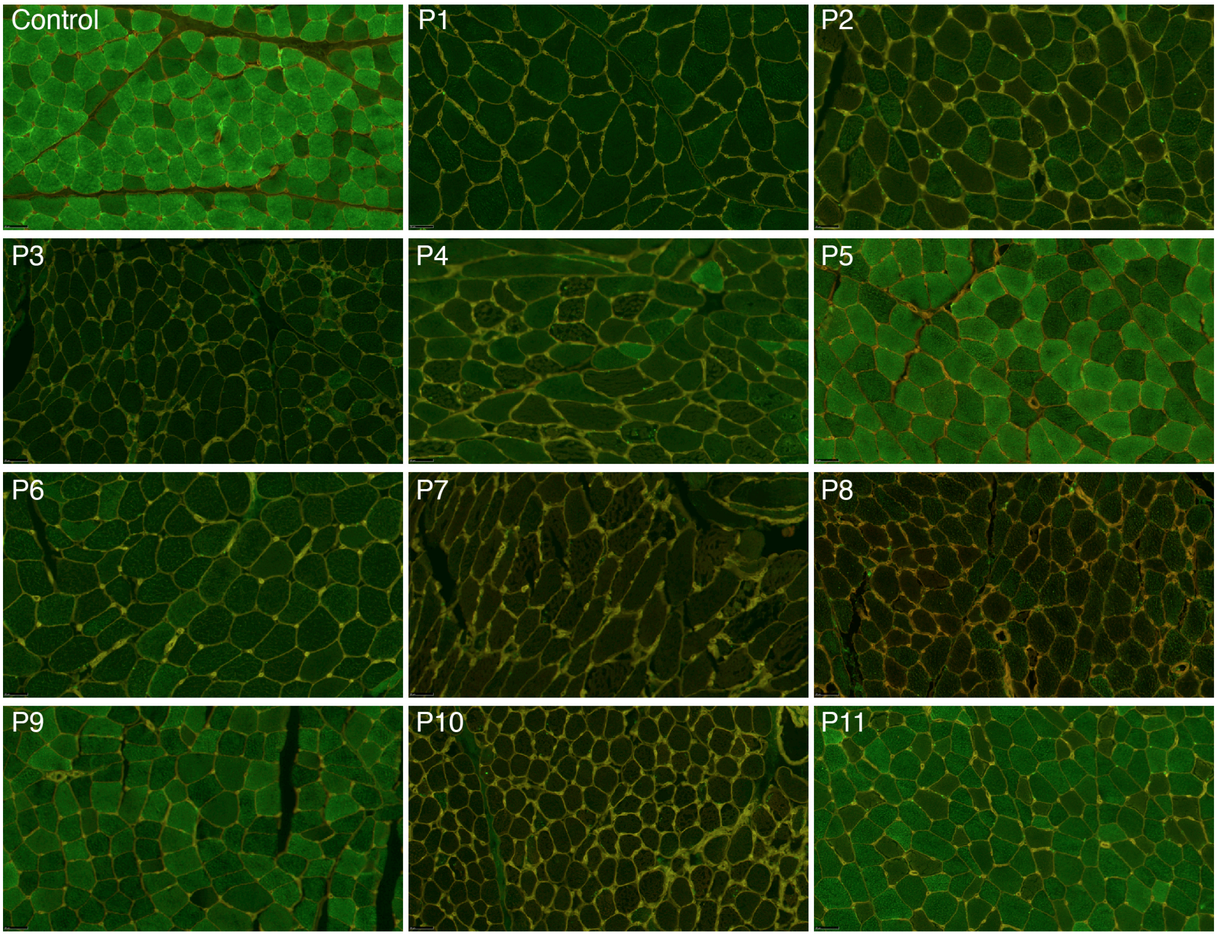

Supplementary Fig 4. Complex I activity (NDUFB8 immunofluorescence, green colour) in single muscle fibers. One control and 11 patients (P1-11). The Complex I immunofluorescence is reduced in all patients. Yellow color corresponds to perlecan, a basement membrane protein.

Supplementary Table 4. List of proteins detected in subunits and assembly factors for complex I – V in the proteomic analysis

Gene Symbol	UniProt Accession	Description	Unique Peptides	log2 FC	FDR
**CI subunits**					
NDUFV3	P56181	NADH dehydrogenase [ubiquinone] flavoprotein 3, mitochondrial	2	-1.88	0.00
NDUFC1	O43677	NADH dehydrogenase [ubiquinone] 1 subunit C1, mitochondrial	2	-1.69	0.00
NDUFS6	O75380	NADH dehydrogenase [ubiquinone] iron-sulfur protein 6, mitochondrial	7	-1.65	0.00
NDUFA7	O95182	NADH dehydrogenase [ubiquinone] 1 alpha subcomplex subunit 7	4	-1.64	0.00
NDUFB4	O95168	NADH dehydrogenase [ubiquinone] 1 beta subcomplex subunit 4	4	-1.63	0.00
NDUFS4	O43181	NADH dehydrogenase [ubiquinone] iron-sulfur protein 4, mitochondrial	3	-1.63	0.00
NDUFA2	O43678	NADH dehydrogenase [ubiquinone] 1 alpha subcomplex subunit 2	5	-1.62	0.00
MT-ND4	P03905	NADH-ubiquinone oxidoreductase chain 4	1	-1.61	0.00
NDUFA10	O95299	NADH dehydrogenase [ubiquinone] 1 alpha subcomplex subunit 10, mitochondrial	14	-1.6	0.00
NDUFA11	Q86Y39	NADH dehydrogenase [ubiquinone] 1 alpha subcomplex subunit 11	5	-1.58	0.00
NDUFA9	Q16795	NADH dehydrogenase [ubiquinone] 1 alpha subcomplex subunit 9, mitochondrial	16	-1.57	0.00
NDUFB8	O95169	NADH dehydrogenase [ubiquinone] 1 beta subcomplex subunit 8, mitochondrial	4	-1.56	0.00
NDUFA12	Q9UI09	NADH dehydrogenase [ubiquinone] 1 alpha subcomplex subunit 12	7	-1.56	0.00
NDUFB7	P17568	NADH dehydrogenase [ubiquinone] 1 beta subcomplex subunit 7	6	-1.56	0.00
NDUFV1	P49821	NADH dehydrogenase [ubiquinone] flavoprotein 1, mitochondrial	15	-1.51	0.00
NDUFS1	P28331	NADH-ubiquinone oxidoreductase 75 kDa subunit, mitochondrial	35	-1.5	0.00
NDUFS5	O43920	NADH dehydrogenase [ubiquinone] iron-sulfur protein 5	7	-1.48	0.00
NDUFB9	Q9Y6M9	NADH dehydrogenase [ubiquinone] 1 beta subcomplex subunit 9	6	-1.48	0.00
NDUFB3	O43676	NADH dehydrogenase [ubiquinone] 1 beta subcomplex subunit 3	4	-1.42	0.00
NDUFB1	O75438	NADH dehydrogenase [ubiquinone] 1 beta subcomplex subunit 1	2	-1.41	0.00
NDUFA13	Q9P0J0	NADH dehydrogenase [ubiquinone] 1 alpha subcomplex subunit 13	5	-1.40	0.00
NDUFC2	O95298	NADH dehydrogenase [ubiquinone] 1 subunit C2	5	-1.40	0.00
NDUFB6	O95139	NADH dehydrogenase [ubiquinone] 1 beta subcomplex subunit 6	4	-1.36	0.00
NDUFA8	P51970	NADH dehydrogenase [ubiquinone] 1 alpha subcomplex subunit 8	6	-1.32	0.00
NDUFB10	O96000	NADH dehydrogenase [ubiquinone] 1 beta subcomplex subunit 10	6	-1.27	0.00
NDUFB5	O43674	NADH dehydrogenase [ubiquinone] 1 beta subcomplex subunit 5, mitochondrial	5	-1.27	0.00
NDUFV2	P19404	NADH dehydrogenase [ubiquinone] flavoprotein 2, mitochondrial	9	-1.26	0.00
NDUFA6	P56556	NADH dehydrogenase [ubiquinone] 1 alpha subcomplex subunit 6	4	-1.19	0.00
NDUFB11	Q9NX14	NADH dehydrogenase [ubiquinone] 1 beta subcomplex subunit 11, mitochondrial	3	-1.10	0.00
MT-ND3	P03897	NADH-ubiquinone oxidoreductase chain 3	1	-1.04	0.01
MT-ND5	P03915	NADH-ubiquinone oxidoreductase chain 5	1	-0.84	0.00
NDUFS2	O75306	NADH dehydrogenase [ubiquinone] iron-sulfur protein 2, mitochondrial	13	-0.82	0.00
NDUFS3	O75489	NADH dehydrogenase [ubiquinone] iron-sulfur protein 3, mitochondrial	7	-0.82	0.00
NDUFS7	O75251	NADH dehydrogenase [ubiquinone] iron-sulfur protein 7, mitochondrial	4	-0.79	0.00
NDUFA5	Q16718	NADH dehydrogenase [ubiquinone] 1 alpha subcomplex subunit 5	4	-0.78	0.00
NDUFS8	O00217	NADH dehydrogenase [ubiquinone] iron-sulfur protein 8, mitochondrial	5	-0.75	0.00
NDUFAB1	O14561	Acyl carrier protein, mitochondrial	2	0.20	0.4
**CI assembly factors**					
AIFM1	O95831	Apoptosis-inducing factor 1, mitochondrial	21	0.14	0.44
NUBPL	Q8TB37	Iron-sulfur cluster transfer protein NUBPL	4	0.14	0.70
TMEM126 A	Q9H061	Transmembrane protein 126A OS=Homo sapiens	5	0.30	0.33
NDUFAF7	Q7L592	Protein arginine methyltransferase NDUFAF7, mitochondrial	1	0.51	0.11
NDUFAF5	Q5TEU4	Arginine-hydroxylase NDUFAF5, mitochondrial	2	0.74	0.01
ACAD9	Q9H845	Complex I assembly factor ACAD9, mitochondrial	8	0.78	0.01
NDUFAF2	Q8N183	NADH dehydrogenase [ubiquinone] 1 alpha subcomplex assembly factor 2	8	0.92	0.01
NDUFAF6	Q330K2	NADH dehydrogenase (ubiquinone) complex I, assembly factor 6	3	0.92	0.01
TMEM70	Q9BUB7	Transmembrane protein 70, mitochondrial	4	0.97	0.02
NDUFAF1	Q9Y375	Complex I intermediate-associated protein 30, mitochondrial	1	0.97	0.00
COA1	Q9GZY4	Cytochrome c oxidase assembly factor 1 homolog	1	0.99	0.01
NDUFAF3	Q9BU61	NADH dehydrogenase [ubiquinone] 1 alpha subcomplex assembly factor 3	5	1.22	0.00
ECSIT	Q9BQ95	Evolutionarily conserved signaling intermediate in Toll pathway, mitochondrial	1	1.55	0.00
NDUFAF4	Q9P032	NADH dehydrogenase [ubiquinone] 1 alpha subcomplex assembly factor 4	8	1.57	0.00
TIMMDC1	Q9NPL8	Complex I assembly factor TIMMDC1, mitochondrial	2	2.02	0.02

**CII subunits**					
SDHD	O14521	Succinate dehydrogenase [ubiquinone] cytochrome b small subunit, mitochondrial	2	-0.69	0.03
SDHA	P31040	Succinate dehydrogenase [ubiquinone] flavoprotein subunit, mitochondrial	25	-0.62	0.01
SDHB	P21912	Succinate dehydrogenase [ubiquinone] iron-sulfur subunit, mitochondrial	15	-0.61	0.01
SDHC	Q99643	Succinate dehydrogenase cytochrome b560 subunit, mitochondrial	2	-0.58	0.01

**CIII subunits**					
MT-CYB	P00156	Cytochrome b	1	-0.02	0.89
UQCRFS1	P47985	Cytochrome b-c1 complex subunit Rieske, mitochondrial	8	0.13	0.45
UQCRC2	P22695	Cytochrome b-c1 complex subunit 2, mitochondrial	22	0.16	0,.36
UQCRC1	P31930	Cytochrome b-c1 complex subunit 1, mitochondrial	18	0.17	0.29
UQCR10	Q9UDW1	Cytochrome b-c1 complex subunit 9	1	0.17	0.40
UQCRQ	O14949	Cytochrome b-c1 complex subunit 8	4	0.17	0.31
UQCRB	P14927	Cytochrome b-c1 complex subunit 7	6	0,19	0.28
CYC1	P08574	Cytochrome c1, heme protein, mitochondrial	10	0.46	0.03
UQCRH	P07919	Cytochrome b-c1 complex subunit 6, mitochondrial	4	0.89	0.00
**CIII assembly factors**					
LYRM7	Q5U5X0	Complex III assembly factor LYRM7	3	0.02	0.94
UQCC2	Q9BRT2	Ubiquinol-cytochrome c reductase complex assembly factor 2	2	0.95	0.00
BCS1L	Q9Y276	Mitochondrial chaperone BCS1	4	1.14	0.00
UQCC1	Q9NVA1	Ubiquinol-cytochrome c reductase complex assembly factor 1	8	1.17	0.00
**CIV subunits**					
MT-CO3	P00414	Cytochrome c oxidase subunit 3	1	-0.75	0.11
COX7A1	P24310	Cytochrome c oxidase subunit 7A1, mitochondrial	2	-0.71	0.01
COX7B	P24311	Cytochrome c oxidase subunit 7B, mitochondrial	1	-0.70	0.01
COX7C	P15954	Cytochrome c oxidase subunit 7C, mitochondrial	2	-0.63	0.01
COX6C	P09669	Cytochrome c oxidase subunit 6C	4	-0.62	0.01
COX6B1	P14854	Cytochrome c oxidase subunit 6B1	6	-0.52	0.03
COX5B	P10606	Cytochrome c oxidase subunit 5B, mitochondrial	7	-0.51	0.02
MT-CO2	P00403	Cytochrome c oxidase subunit 2	7	-0.51	0.02
NDUFA4	O00483	Cytochrome c oxidase subunit NDUFA4	5	-0.44	0.02
COX4I1	P13073	Cytochrome c oxidase subunit 4 isoform 1, mitochondrial	11	-0.44	0.03
COX5A	P20674	Cytochrome c oxidase subunit 5A, mitochondrial	7	-0.37	0.08
COX7A2	P14406	Cytochrome c oxidase subunit 7A2, mitochondrial	2	0.01	0.99
COX7A2L	O14548	Cytochrome c oxidase subunit 7A-related protein, mitochondrial	2	0.19	0.51
**CIV assembly factors**					
PET100	P0DJ07	Protein PET100 homolog, mitochondrial	2	-0.02	0.94
CMC1	Q7Z7K0	COX assembly mitochondrial protein homolog	1	0.03	0.91
COA6	Q5JTJ3	Cytochrome c oxidase assembly factor 6 homolog	2	0.10	0.49
COA3	Q9Y2R0	Cytochrome c oxidase assembly factor 3 homolog, mitochondrial	4	0.29	0.18
CMC2	Q9NRP2	COX assembly mitochondrial protein 2 homolog	1	0.38	0.14
COX19	Q49B96	Cytochrome c oxidase assembly protein COX19	3	0.38	0.00
COX11	Q9Y6N1	Cytochrome c oxidase assembly protein COX11, mitochondrial	3	0.58	0.01
TIMM21	Q9BVV7	Mitochondrial import inner membrane translocase subunit Tim21	5	0.62	0.01
TACO1	Q9BSH4	Translational activator of cytochrome c oxidase 1	4	0.68	0.01
COX14	Q96I36	Cytochrome c oxidase assembly protein COX14	1	0.74	0.02
COA7	Q96BR5	Cytochrome c oxidase assembly factor 7	4	0.81	0.00
SMIM20	Q8N5G0	Small integral membrane protein 20	1	0.84	0.03
SCO1	O75880	Protein SCO1 homolog, mitochondrial	2	0.87	0.01
SCO2	O43819	Protein SCO2 homolog, mitochondrial	3	0.98	0.01
COX20	Q5RI15	Cytochrome c oxidase assembly protein COX20, mitochondrial	2	1.14	0.01
COX15	Q7KZN9	Cytochrome c oxidase assembly protein COX15 homolog	2	1.34	0.01
SURF1	Q15526	Surfeit locus protein 1	3	1.55	0.00
**CV subunits**					
DMAC2L	Q99766	ATP synthase subunit s, mitochondrial	7	0.49	0.03
ATP5MG	O75964	ATP synthase subunit g, mitochondrial	7	0.60	0.01
MT-ATP6	P00846	ATP synthase subunit a	2	0.62	0.01
ATP5F1D	P30049	ATP synthase subunit delta, mitochondrial	3	0.68	0.00
ATP5ME	P56385	ATP synthase subunit e, mitochondrial	5	0.71	0.00
ATP5PB	P24539	ATP synthase F(0) complex subunit B1, mitochondrial	13	0.73	0.00
ATP5MF	P56134	ATP synthase subunit f, mitochondrial	2	0.74	0.00
ATP5PD	O75947	ATP synthase subunit d, mitochondrial	11	0.75	0.00
ATP5MC3	P48201	ATP synthase F(0) complex subunit C3, mitochondrial	1	0.77	0.00
ATP5F1B	P06576	ATP synthase subunit beta, mitochondrial	25	0.78	0.00
ATP5PO	P48047	ATP synthase subunit O, mitochondrial	10	0.79	0.00
ATP5F1A	P25705	ATP synthase subunit alpha, mitochondrial	36	0.79	0.00
ATP5F1C	P36542	ATP synthase subunit gamma, mitochondrial	12	0.84	0.00
MT-ATP8	P03928	ATP synthase protein 8	1	0.94	0.01
ATP5F1E	P56381	ATP synthase subunit epsilon, mitochondrial	3	0.98	0.00
ATP5IF1	Q9UII2	ATPase inhibitor, mitochondrial	4	1.16	0,01
ATP5PF	P18859	ATP synthase-coupling factor 6, mitochondrial	4	1.18	0.00
**CV assembly factors**					
ATPAF2	Q8N5M1	ATP synthase mitochondrial F1 complex assembly factor 2	5	0.60	0.03
FMC1	Q96HJ9	Protein FMC1 homolog	2	0.86	0.02
ATPAF1	Q5TC12	ATP synthase mitochondrial F1 complex assembly factor 1	5	0.93	0.01

FDR values marked in grey are not significant (P>0.05); C, complex

Supplementary Table 5. List of proteins detected in fatty acid oxidation and citric acid cycle in the proteomic analysis

Gene Symbol	UniProt Accession	Description	Unique Peptides	log2 FC	FDR
**Fatty Acid Oxidation (FAO)**					
ACADS	P16219	Short-chain specific acyl-CoA dehydrogenase, mitochondrial	12	-1.99	0.01
ETFDH	Q16134	Electron transfer flavoprotein-ubiquinone oxidoreductase, mitochondrial	15	-1.08	0.00
ACADSB	P45954	Short/branched chain specific acyl-CoA dehydrogenase, mitochondrial	7	-1.04	0.01
ACADM	P11310	Medium-chain specific acyl-CoA dehydrogenase, mitochondrial	15	-0.88	0.05
ACSS1	Q9NUB1	Acetyl-coenzyme A synthetase 2-like, mitochondrial	9	-0.08	0.84
ACOT11	Q8WXI4	Acyl-coenzyme A thioesterase 11	1	0.25	0.60
ACSF2	Q96CM8	Medium-chain acyl-CoA ligase ACSF2, mitochondrial	11	0.28	0.02
CROT	Q9UKG9	Peroxisomal carnitine O-octanoyltransferase	1	0.28	0.54
ACADVL	P49748	Very long-chain specific acyl-CoA dehydrogenase, mitochondrial	37	0.36	0.25
CRAT	P43155	Carnitine O-acetyltransferase	23	0.41	0.03
ECHS1	P30084	Enoyl-CoA hydratase, mitochondrial	16	0.54	0.01
MMUT	P22033	Methylmalonyl-CoA mutase, mitochondrial	8	0.56	0.03
ACAT1	P24752	Acetyl-CoA acetyltransferase, mitochondrial	22	0.58	0.03
ECI1	P42126	Enoyl-CoA delta isomerase 1, mitochondrial	8	0.61	0.01
ACACB	O00763	Acetyl-CoA carboxylase 2 O	10	0.74	0.00
DECR1	Q16698	2,4-dienoyl-CoA reductase [(3E)-enoyl-CoA-producing], mitochondrial	14	0.89	0.00
HADH	Q16836	Hydroxyacyl-coenzyme A dehydrogenase, mitochondrial	12	0.94	0.01
MCEE	Q96PE7	Methylmalonyl-CoA epimerase, mitochondrial	5	1.00	0.01
ETFB	P38117	Electron transfer flavoprotein subunit beta	16	1.10	0.00
SLC25A20	O43772	Mitochondrial carnitine/acylcarnitine carrier protein	8	1.03	0.01
HSD17B10	Q99714	3-hydroxyacyl-CoA dehydrogenase type-2	10	1.04	0.00
CPT1B	Q92523	Carnitine O-palmitoyltransferase 1, muscle isoform	23	1.13	0.01
ETFA	P13804	Electron transfer flavoprotein subunit alpha, mitochondrial	11	1.14	0.00
ECI2	O75521	Enoyl-CoA delta isomerase 2	10	1.16	0.00
CPT2	P23786	Carnitine O-palmitoyltransferase 2, mitochondrial	23	1.17	0.01
ACSL1	P33121	Long-chain-fatty-acid--CoA ligase 1	35	1.20	0.00
PCCA	P05165	Propionyl-CoA carboxylase alpha chain, mitochondrial	18	1.31	0.01
PCCB	P05166	Propionyl-CoA carboxylase beta chain, mitochondrial	12	1.33	0.01
ACAA2	P42765	3-ketoacyl-CoA thiolase, mitochondrial	21	1.34	0.00
HADHB	P55084	Trifunctional enzyme subunit beta, mitochondrial	27	1.73	0,00
HADHA	P40939	Trifunctional enzyme subunit alpha, mitochondrial	40	1.78	0.00
CPT1A	P50416	Carnitine O-palmitoyltransferase 1, liver isoform	4	2.17	0.00
**Citric acid cycle (TCA)**					
DLD	P09622	Dihydrolipoyl dehydrogenase, mitochondrial	14	-1.15	0.01
OGDH	Q02218	2-oxoglutarate dehydrogenase complex component E1	42	-0.49	0.03
PC	P11498	Pyruvate carboxylase, mitochondrial	1	0.35	0.03
SLC25A11	Q02978	Mitochondrial 2-oxoglutarate/malate carrier protein	13	0.37	0.03
L2HGDH	Q9H9P8	L-2-hydroxyglutarate dehydrogenase, mitochondrial	7	0.05	0.80
ACLY	P53396	ATP-citrate synthase	6	0.26	0.29
ME2	P23368	NAD-dependent malic enzyme, mitochondrial	4	0.40	0.07
IDH2	P48735	Isocitrate dehydrogenase [NADP], mitochondrial	26	0.51	0.01
SUCLA2	Q9P2R7	Succinate--CoA ligase [ADP-forming] subunit beta, mitochondrial	23	0.52	0.01
MPC2	O95563	Mitochondrial pyruvate carrier 2	1	0.62	0.14
MDH2	P40926	Malate dehydrogenase, mitochondrial	19	0.64	0.01
MPC1	Q9Y5U8	Mitochondrial pyruvate carrier 1	1	0.69	0.04
DLST	P36957	Dihydrolipoyllysine-residue succinyltransferase component of 2-oxoglutarate	14	0.73	0.01
SUCLG1	P53597	Succinate--CoA ligase [ADP/GDP-forming] subunit alpha, mitochondrial	10	0.76	0.00
ABHD11	Q8NFV4	sn-1-specific diacylglycerol lipase ABHD11	3	0.79	0.00
CS	O75390	Citrate synthase, mitochondrial	16	0.79	0.00
FAHD1	Q6P587	Acylpyruvase FAHD1, mitochondrial	7	0.85	0.00
IDH3B	O43837	Isocitrate dehydrogenase [NAD] subunit beta, mitochondrial	11	0.88	0.02
FH	P07954	Fumarate hydratase, mitochondrial	20	0.90	0.00
ACO2	Q99798	Aconitate hydratase, mitochondrial	41	0.94	0.00
IDH3G	P51553	Isocitrate dehydrogenase [NAD] subunit gamma, mitochondrial	9	0.97	0.01
IDH3A	P50213	Isocitrate dehydrogenase [NAD] subunit alpha, mitochondrial	16	0.99	0.01
ME3	Q16798	NADP-dependent malic enzyme, mitochondrial	5	1.00	0.00
SUCLG2	Q96I99	Succinate--CoA ligase [GDP-forming] subunit beta, mitochondrial	19	1.56	0.00
PCK2	Q16822	Phosphoenolpyruvate carboxykinase [GTP], mitochondrial	8	3.10	0.00

FDR values marked in grey are not significant (P>0.05)

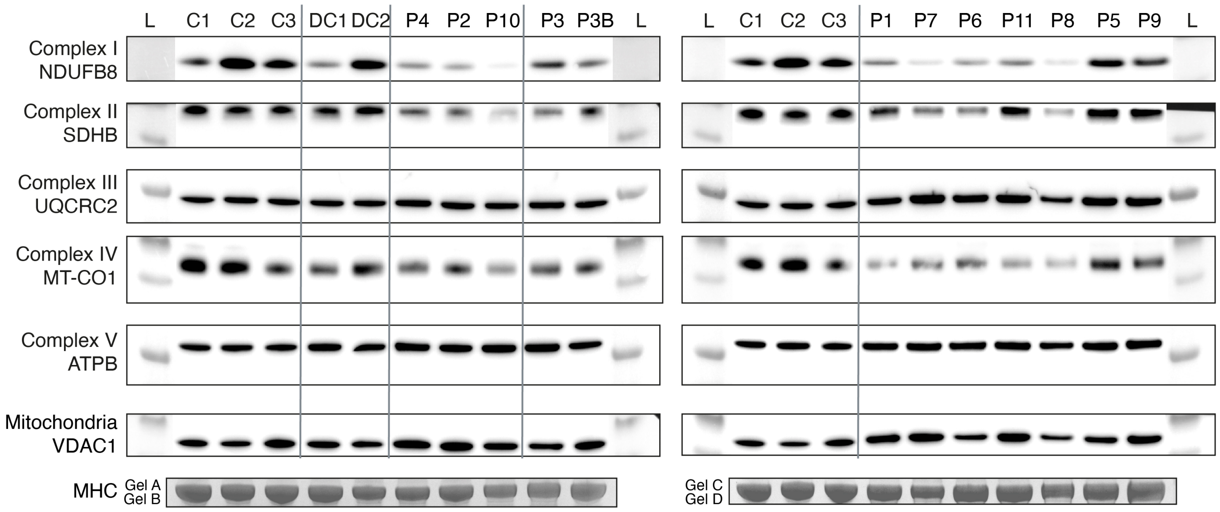

Supplementary Fig 5. Western blot analysis. The overall pattern showed a reduced amount of Complex I, II and IV, but no reduction of Complex III and V. The membranes were cut into pieces according to the size of the corresponding proteins to be able to incubate several antibodies on one blotted membrane. This was done for antibodies that are well-known to only show a band of correct molecular size. The figure of the western blot result is arranged from four agarose gels with equal amounts of loaded proteins derived from a single concentration measurement. L, Ladder; C, control; DC, disease control; (C and DC are specified in Supplementary Table 2); P, patients; MHC, Myosin heavy chain, Gel A and C, antibodies for ATPB, MT-CO1, NDUFB8, Gel B and D antibodies for UQCRC1, VDAC1, SDHB

Pharmocogenetic analysis

Sertraline is metabolized by CYP enzymes and pharmocogenetic studies suggest that *CYP2C19* is the major metabolic enzyme. Since some variants are reported to affect the enzyme activity we analyzed the presence of these variants in our patients (star alleles *). We included 11 variants in our analysis (Supplementary Table 6). We identified two of the variants in 6 of our patients, CYP2C19*2 detected in 4 and CYP2C19*17 in 2. Both variants are common in the population with more than 20 000 homozygous individuals detected for each variant in the gnomAD database.

Supplementary Table 6. Genetic analyses of individual Star alleles* for CYP2C19 with effect on enzyme activity

Allele	cDNA	Protein change	Effect on enzyme activity	dbSNP rsID	gnomAD v4.1.0	11 patients 22 alleles
**1*	None (wt)		Normal (extensive) activity			10/11<br>15/22
**2*	c.681G>A	p.P227P	No activity	rs4244285	234686/1464646[a]<br>1.60e-1[b]<br>20742[c]	4/11<br>5/22[d]
**3*	c.636G>A	p.W212*	No activity	rs4986893	4750/1613588[a]<br>0.002944[b]<br>214[c]	0/22
**4*	c.1A>G	p.M1?	No activity	rs28399504	3866/1613130[a]<br>2.40e-3[b]<br>16[c]	0/22
**5*	c.1297C>T	p.R433W	No activity	rs56337013	28/1613634[a]<br>1.74e-5[b]<br>0[c]	0/22
**6*	c.395G>A	p.R132Q	No activity	rs72552267	489/1614044[a]<br>3.03e-4[b]<br>1[c]	0/22
**7*	c.819+2T>A	splice	No activity	rs72558186	3/1535118[a]<br>1.95e-6[b]<br>0[c]	0/22
**8*	c.358T>C	p.W120R	No activity	rs41291556	3867/1614036[a]<br>2.40e-3[b]<br>12[c]	0/22
**9*	c.431G>A	p.R144H	Decreased activity	rs17884712	1141/1614010[a]<br>7.07e-4[b]<br>7[c]	0/22
**10*	c.680C>T	p.P227L	Decreased activity	rs6413438	354/1471456[a]<br>2.41e-4[b]<br>1[c]	0/22
**17*	c.-806C>T	promotor	Enhanced activity	rs12248560	30385/151940[a]<br>0.2000[b]<br>3261[c]	2/11<br>2/22
**35*	c.332-23A>G[e]	splice	No activity	rs12769205	266140/1612890[a]<br>1.65e-1[b]<br>24492[c]	0/22

Included Star alleles* based on https://www.mayocliniclabs.com/test-catalog/overview/610043#Clinical-and-Interpretive, *CYP2C19*; NM_000769.1

[a] allele count/total allele number, [b] allele frequency, [c] number of homozygous, [d] together with c.332-23A>G,
[e] in the absence of c.681G>A
